# Characterization of Single Gene Deletion Mutants Affecting Alternative Oxidase Production in *Neurospora crassa*: Role of the *yvh1* Gene

**DOI:** 10.3390/microorganisms8081186

**Published:** 2020-08-04

**Authors:** Adrien Beau Desaulniers, Nishka Kishore, Kelly Adames, Frank E. Nargang

**Affiliations:** Department of Biological Sciences, University of Alberta, Edmonton, AB T6G 2E9, Canada; beau.des@gmail.com (A.B.D.); kishore@ualberta.ca (N.K.); kelly.adames@ualberta.ca (K.A.)

**Keywords:** *Neurospora crassa*, mitochondria, alternative oxidase, YVH1

## Abstract

The *Neurospora crassa* AOD1 protein is a mitochondrial alternative oxidase that passes electrons directly from ubiquinol to oxygen. The enzyme is encoded by the nuclear *aod-1* gene and is produced when the standard electron transport chain is inhibited. We previously identified eleven strains in the *N. crassa* single gene deletion library that were severely deficient in their ability to produce AOD1 when grown in the presence of chloramphenicol, an inhibitor of mitochondrial translation that is known to induce the enzyme. Three mutants affected previously characterized genes. In this report we examined the remaining mutants and found that the deficiency of AOD1 was due to secondary mutations in all but two of the strains. One of the authentic mutants contained a deletion of the *yvh1* gene and was found to have a deficiency of *aod-1* transcripts. The YVH1 protein localized to the nucleus and a post mitochondrial pellet from the cytoplasm. A zinc binding domain in the protein was required for rescue of the AOD1 deficiency. In other organisms YVH1 is required for ribosome assembly and mutants have multiple phenotypes. Lack of YVH1 in *N. crassa* likely also affects ribosome assembly leading to phenotypes that include altered regulation of AOD1 production.

## 1. Introduction

The proteins found in mitochondria are encoded on two genomes. The majority of genes encoding mitochondrial proteins are found in the nuclear DNA, while a small number are found in mtDNA. To achieve proper levels of expression from each of the genomes, signaling must occur between mitochondria and the nucleus. Mitochondria communicate their status to the nucleus to stimulate changes in expression of appropriate nuclear encoded mitochondrial proteins by a process called retrograde regulation, which has been shown to be an important pathway in many different species [1,2,3,4,5].

The alternative oxidase (AOX) is an example of a mitochondrial enzyme that is encoded in the nucleus. It exists as a homodimeric interfacial membrane protein with domains that are inserted into the inner leaflet of the mitochondrial inner membrane [6,7,8]. Genes encoding the enzyme occur in many species, though AOX has not been found in higher animals [9,10,11,12]. The AOX provides a path of electron flow that is an alternative to the standard electron transport chain (sETC). Following movement of electrons via Complex I to ubiquinol, the AOX transfers the electrons directly to molecular oxygen, bypassing Complexes III and IV of the sETC [10,13,14,15,16]. In many organisms, AOX is not expressed under standard growth conditions. However, a number of factors that affect mitochondrial function induce transcription of AOX genes and the synthesis of the enzyme [10,13,14,15,16,17]. Since expression of the nuclear encoded AOX responds to changes in mitochondrial function, it serves as a robust model for the study of retrograde regulation.

Our laboratory has studied the expression of AOX in the filamentous fungus *Neurospora crassa*, which contains two different structural genes for the enzyme called *aod-1* and *aod-3* [18,19]. In standard growth conditions the *aod-1* gene is transcribed at very low levels and the AOD1 protein is not detectable. However, expression of *aod-1* is induced when cells are grown in the presence of inhibitors such as antimycin A (AntA) or cyanide, which affect complex III and complex IV, respectively, of the sETC. Induction of *aod-1* transcription also leads to the appearance of the AOD1 protein [19,20,21,22,23]. A similar effect is seen in strains containing mutations that affect the function or synthesis of proteins found in the complexes of the sETC [18,23,24,25]. Growth in the presence of drugs such as chloramphenicol (Cm), which inhibit mitochondrial translation, also induce expression of *aod-1* because they reduce the amount of mitochondrially encoded proteins that are found in sETC complexes [15,20,26,27]. Unlike *aod-1*, no conditions have been found that result in transcription of *aod-3* [19].

A number of genes that affect *aod-1* expression have been identified [23,28,29]. Two zinc cluster transcription factors, encoded by the *aod-2* and *aod-5* genes [23,30], bind constitutively as a heterodimer in the upstream region of the *aod-1* gene and are required for the increased transcription of *aod-1* that occurs under inducing conditions [19,30,31,32,33]. Similarly, the expression of AOX in *Aspergillus nidulans* and *Podospora anserina* requires the orthologues of *N. crassa* AOD2 and AOD5 [34,35,36]. In all three species, these transcription factors play additional roles in the expression of certain genes involved in gluconeogenesis and other metabolic processes [33,35,36].

We have also demonstrated that the expression of *aod-1* is influenced by a G protein signaling pathway. Strains lacking the FLBA protein have high levels of *aod-1* transcript, even when grown in standard medium lacking any inducing drugs [37]. FLBA is a regulator of G protein signaling. Its function is to stimulate the relatively slow intrinsic GTPase activity of G_α_ that converts the active G_α_-GTP complex to the inactive G_α_-GDP complex. Thus, in *flba* mutants, the active G_α_-GTP complex persists for longer times. Interestingly, even though relatively high levels of *aod-1* transcript are present in strains lacking FLBA, the levels of AOD1 protein remain virtually undetectable. However, growth in the presence of typical inducing agents does result in the appearance of the AOD1 protein in *flba* mutants. These observations were interpreted as evidence for a control system that prevents translation of the *aod-1* mRNA under non-inducing conditions [37].

In an attempt to identify additional genes required for AOD1 production, we screened the *N. crassa* single gene deletion library [38] for strains that inefficiently expressed AOD1 under inducing conditions [29]. The strains in the deletion library were first examined for their ability to grow in the presence of AntA at a concentration that completely inhibited complex III of the sETC. Under these conditions, wild type strains that produce AOD1 are able to continue growth. However, mutant strains incapable of producing AOD1 are unable to grow in the presence of AntA. Non-growing strains, and strains that grew very slowly in the presence of AntA, were then examined for the presence of AOD1 following growth in the presence of Cm. The concentration of Cm used in such studies inhibits, but does not totally abolish, mitochondrial translation. Thus, the level of key components of the sETC complexes are severely reduced but not totally eliminated. This leads to a reduced level of electron transport via the sETC and results in the induction of the *aod-1* gene and synthesis of the AOD1 protein in wild type strains. However, since mitochondrial translation is not totally eliminated, mutant strains unable to produce AOD1 can still grow via the remaining sETC function. Mitochondria can be isolated from such strains and examined for their level of AOD1 on western blots.

The screen of the deletion library identified mutants that were placed into three different classes based on the level of AOD1 detected in mitochondria following growth in the presence of Cm. Class 1 mutants were defined as those lacking detectable levels of AOD1 after growth in the presence of Cm for 24 h, or 48 h, or both periods of growth. The class 2 and class 3 mutants had reduced, but detectable, levels of AOD1 following growth in the presence of Cm [29]. Of the eleven class 1 mutants identified in the deletion library, three were found to carry deletions of the previously characterized *aod-1*, *aod-2*, and *aod-5* genes. In the present study we have analyzed the remaining eight class 1 mutants previously identified, plus a ninth strain identified after publication of the previous study. Surprisingly, the AOD1 deficiency in seven of the nine strains was found to be due to random secondary mutations that were either created during the procedure used to generate the targeted deletions or preexisted in the transformed conidia. Thus, the designed deletion was responsible for the AOD1 deficiency in only two of the nine strains. One strain carries a deletion of a gene encoding a ubiquinone biosynthesis protein. A preliminary characterization of that strain was performed previously [29]. In the present study we analyzed the remaining class 1 mutant. The strain carries a deletion of the *yvh1* gene. The YVH1 protein is known to be involved in ribosome assembly in other systems, and loss of the functional protein results in an array of phenotypes [39,40,41,42,43]. However, to our knowledge, no previous studies have examined the effect of *yvh1* mutations on AOX expression.

## 2. Materials and Methods

### 2.1. Growth and Handling of N. crassa

Table 1 lists the strains used in this study. Unless otherwise stated, growth and handling of the *N. crassa* strains were performed using media and techniques described previously [44]. To produce conidia, most strains were grown in the dark at 30 °C in 250 mL Erlenmeyer flasks containing 50 mL of agar solidified Vogel’s salts [45] with sucrose as the carbon source and with trace elements and biotin added (VSuTB) for 48 h. Flasks were then moved to room temperature with constant light to allow formation of conidia. The Δ*yvh1* strain and strains containing mutations in the zinc-binding domain of the *yvh1* gene were deficient in conidiation. They were grown at 30 °C in 125 mL Erlenmeyer flasks containing 25 mL of solid agar VSuTB for 72 h before being moved to room temperature with light.

When required, the following inhibitors were added to liquid or solid media at the final concentrations shown in parentheses: Cm (2 mg/mL); AntA (0.5 μg/mL); basta, also known as glufosinate or phosphinothricin, (200 μg/mL); bleomycin (1.0 μg/mL); hygromycin B (Hyg, 200 μg/mL).

### 2.2. Measurement of Growth Rates

Conidia from the *N. crassa* strains being investigated were harvested in 50 mL of sterile distilled water and centrifuged in a clinical centrifuge (International Equipment Company, Needham Heights, MA, USA) at maximum speed for 2 min to pellet the conidia. Conidia were washed once in sterile distilled water, resuspended in 1 mL of sterile, distilled water and transferred to a 1.5 mL Eppendorf tube. The concentration of conidia was determined using a hemocytometer. Suspensions with final concentrations of 10^7^, 10^6^, and 10^5^ conidia/mL were prepared and 10 μL of each dilution was spotted on plates containing standard Vogel’s sorbose medium (VSoTB), to induce colony formation, with or without inhibitors. Unless specified otherwise, plates without inhibitors were incubated for 48 h at 30 °C. Plates containing inhibitors were incubated for 68 h.

### 2.3. Construction of Plasmids Used for Gene Rescue and Isolation of Transformants

Plasmids for gene rescue experiments were constructed by standard cloning methods using restriction enzymes, generation of specific DNA products by PCR, ligation of DNA fragments in vitro, and site directed mutagenesis [46]. For the rescue of strain 100B5, which carries a deletion of the NCU08158 gene, the base of the rescue plasmid was pBARKS1. This plasmid carries genes for resistance to ampicillin and basta [47]. The 1314 bp gene for NCU08158, plus 617 bp upstream from the start codon and 518 bp downstream of the stop codon, were cloned into pBARSK1. Coding information for a tag carrying an epitope of hemagglutinin (YPYDVPDYA) in triplicate (3xHA) was added to the coding sequence either just following the start codon (N-terminal tag) or just prior to the stop codon (C-terminal tag) of the gene. Plasmids containing the N- and C-terminal tagged versions of NCU08158 were transformed into strain 100B5 by electroporation of conidia as described previously [48]. Transformed conidia were plated on VSoTB plates containing basta and incubated at 30 °C for three to five days. Basta resistant colonies were picked to VSuTB slants containing basta and allowed to conidiate. Conidia were then streaked onto basta containing plates. Single colonies that formed were then picked to slants with no drugs. These strains were analyzed further for evidence of rescue of the ability to produce AOD1 protein when grown under inducing conditions.

### 2.4. Construction of Plasmids Encoding Mutant Versions of the YVH1 Protein

To create the phosphatase domain mutants (P-mutants or Pm) and the zinc binding domain mutants (Z-mutants or Zm), primers were designed to change the coding sequence of the N-terminal 3xHA tagged *yvh1^+^* gene in the rescuing plasmid at specific regions via site directed PCR mutagenesis. The amino acid changes for the P-mutants were H149Q, C150S, and R156Q. These changes all affect the conserved HC(X)_5_R phosphatase domain of the protein and all were introduced into a single gene. To create the Z-mutants, two cysteine residues of the zinc-binding domain were mutated to serine (C340S and C345S). All mutations were confirmed by DNA sequencing.

### 2.5. Isolation of Mitochondria, Cytosolic, and Post-Mitochondrial Pellet (PMP) Fractions

*N. crassa* cultures were grown for 20 h, unless specified otherwise, at 30 °C with aeration by shaking in liquid VSuTB medium with or without Cm in baffled flasks. Crude mitochondria were isolated as previously described [49] with modifications. Briefly, mycelium was harvested by vacuum filtration and weighed. Samples were ground for one minute using a mortar and pestle with sand (Sigma-Aldrich, St. Louis, MO, USA) equal to the weight of the mycelium, and SEMP (0.25 M sucrose; 10 mM MOPS, pH 7.2; 1 mM EDTA; 1 mM phenylmethylsulphonyl fluoride (PMSF)) isolation buffer (1 mL/g of mycelium) containing protease inhibitors (final concentrations of 2 μg/mL aprotinin, 1 μg/mL leupeptin and 1 μg/mL pepstatin A). After grinding, another volume of SEMP plus protease inhibitors (1 mL/g of mycelium) was added. The slurry was centrifuged at 3600× *g* (5000 rpm) twice for 5 min using an SA-600 rotor in a Sorvall RC-5C Plus centrifuge (Sorvall, Mandel Scientific, Guelph, ON, Canada) keeping the supernatant each time. The supernatant, which contained both cytosol and mitochondria, was then centrifuged at 21,000× *g* (12,000 rpm) for 20 min to pellet mitochondria. If required, the supernatant was saved as a crude cytosolic fraction for further use (see below). The pellet was washed by resuspending in 5 mL of SEMP and the 21,000× *g* centrifugation was repeated. The pellet was resuspended in 1 mL of SEMP, transferred to a microcentrifuge tube, and centrifuged at top speed in an Eppendorf tabletop centrifuge (13,000 rpm). The supernatant was discarded and the mitochondrial pellet was resuspended in SEMP plus protease inhibitor. These mitochondria were classified as “crude” mitochondria.

To obtain more highly purified mitochondria for subcellular fractionation experiments, the mitochondria were further purified through a sucrose gradient using a previously described procedure [50] with modifications. Crude mitochondria were pelleted and resuspended in 1.5 mL of SEMP isolation buffer containing 60% sucrose and transferred to a thick wall polycarbonate ultracentrifuge tube (Beckman Instruments Inc., Palo Alto, CA, USA). 1.5 mL of SEMP with 55% sucrose was layered over the 60% layer, and 0.75 mL of SEMP with 44% sucrose was layered over the 55% layer. The sample was centrifuged at 75,000× *g* (35,000 rpm) for 2 h in a TLA110 rotor in a Beckman Optima^TM^ MAX ultracentrifuge (Beckman Instruments, Palo Alto, CA, USA). The mitochondria were isolated from the interface of the 44% and 55% SEMP layers, placed in an Eppendorf tube, and diluted with 1 mL of SEMP. The sample was then centrifuged for 30 min at top speed (13,000 rpm) using a refrigerated Eppendorf tabletop centrifuge and resuspended in 500 μL of SEMP.

The crude cytosolic fraction, obtained as the supernatant from the crude mitochondria isolation described above, was further purified when required. The crude fraction was centrifuged twice for 30 min at 32,500× *g* (15,000 rpm) in an SA-600 rotor using a Sorvall RC-5C Plus centrifuge. The pellet was discarded each time. The final supernatant was used as the crude cytosolic sample in some experiments. For subcellular fractionations, this fraction was further centrifuged to isolate the cytosolic and PMP fractions. The latter contains fragmented endoplasmic reticulum and other organelles. 1.5 mL of the crude cytosolic fraction was placed in a polyallomer Beckman ultracentrifuge tube (Beckman Instruments Inc., Palo Alto, CA, USA). The sample was centrifuged at 130,000× *g* (48,000 rpm) for 1.5 h in a TLA55 rotor using a Beckman Optima^TM^ MAX ultracentrifuge. 800 μL of the supernatant was collected for the cytosolic fraction and the pellet was resuspended in 200 μL of SEMP plus protease inhibitors as the PMP. All centrifugation steps were performed at 4 °C.

### 2.6. Isolation of Nuclei

The isolation of nuclei was accomplished with a modified version of a previously described protocol [51]. Strains were grown in VSuTB liquid medium at 30 °C and harvested by vacuum filtration. The pad of mycelium was weighed and then ground to a very fine powder with a mortar and pestle in the presence of liquid nitrogen. Two volumes (*v*/*w*) of buffer A (1 M sorbitol, 5% (*w*/*v*) Ficoll 400, 20% (*v*/*v*) glycerol, 5 mM MgCl_2_, 10 mM CaCl_2_, 1% (*v*/*v*) Triton-X-100, pH 7.5) containing 1mM PMSF and protease inhibitors (as described in the previous section) were added per gram of mycelium and the mixture was allowed to thaw at room temperature for 5 min. The homogenate was then passed twice through miracloth (Calbiochem, San Diego, CA, USA) to remove the bulk of the cellular debris. Further debris was removed by centrifugation at 2400× *g* (4000 rpm) twice for 10 min using an SA-600 rotor in a Sorvall RC-5C Plus centrifuge with the supernatant being saved after each spin. To obtain crude nuclei the solution was centrifuged for 50 min at 9000× g (7900 rpm). The supernatant was carefully removed and discarded following the spin. The white pellet was resuspended in ice cold buffer B (1 M sucrose, 50 mM Tris-HCl (pH 7.5), 5 mM MgCl_2_, 10 mM CaCl_2_, 1% (*v*/*v*) Triton X-100) containing 1 mM PMSF and protease inhibitors (as described in the previous section). One ml of buffer B was added per 5 g (*v*/*w*) of the starting mycelial weight. A Percoll:sucrose solution was created by adding 9 parts (*v*/*v*) of Percoll (GE Healthcare, Uppsala, Sweden) to 1 part (*v*/*v*) of 2.5 M sucrose. Two ml of the Percoll:sucrose solution was added to 1 mL of nuclei suspended in Buffer B in a 3.2 mL thick wall polycarbonate ultracentrifuge tube (1 tube per 5 g of starting mycelium) and mixed through gentle pipetting. The tubes were placed in a TLA110 rotor and centrifuged for 45 min at 58,000× *g* (38,000 rpm) in a Beckman Optima^TM^ MAX ultracentrifuge. The nuclei could then be seen as white bands in the middle of the gradient. The bands were removed and combined to a single thick wall polycarbonate tube and centrifuged for 2 h at 100,000× *g* (49,000 rpm) to remove the Percoll. The white nuclei appeared on top of the Percoll pellet. The nuclei were transferred to an Eppendorf tube and washed in suspension buffer (25 mM sucrose, 50 mM Tris-HCl (pH 7.5), 5 mM MgCl, 10 mM CaCl_2_) once by pelleting in an Eppendorf tabletop centrifuge at maximum speed (13,000 rpm). The final pellet was suspended in 100 μL of suspension buffer.

### 2.7. Whole Cell Extracts

Cultures were grown in liquid VSuTB medium for 20 h at 30 °C. Mycelium were harvested by vacuum filtration and 1 g of mycelium was ground with 1 g of sand (Sigma-Aldrich, St. Louis, MO, USA) and 1 mL of SEMP containing 2% sodium dodecyl sulfate (SDS) using a mortar and pestle. Samples were centrifuged for 10 min at 3600× g (5000 rpm) in a SA-600 rotor using a Sorvall RC-5C Plus centrifuge to remove cellular debris and sand. The supernatant was collected and protein concentrations were determined using the BCA-200 protein assay system (Pierce, Rockford, IL, USA), which is tolerant of SDS.

### 2.8. Quantitative Real Time Polymerase Chain Reaction (qPCR)

All qPCR data shown is based on four biological samples each tested using three technical replicates. Cells were grown in liquid VSuTB for specified times at 30 °C with or without Cm. For RNA isolation, mycelium was harvested by vacuum filtration and 100 mg was flash frozen in liquid nitrogen. Total RNA was isolated using the RNeasy Kit from Qiagen following the manufacturer’s instructions for fungi (Qiagen Inc., Santa Clarita, CA, USA). RNA integrity was determined using a BioAnalyzer 2100 nano chip (Agilent Technologies, Mississauga, ON, Canada) following the supplier’s instructions. cDNA was reverse transcribed from RNA (1 μg/reaction) using superscript III (Invitrogen, Carlsbad, CA, USA) following the manufacture’s directions. qPCR (StepOnePlus, Applied Biosystems, Foster City, CA) used the synthesized cDNA and PowerSYBR Green PCR master mix (Applied Biosystems, Foster City, CA, USA) with 5 ng of cDNA template and a primer concentration of 200 nM in a total sample volume of 10 μL in a 96-well reaction plate. Samples were normalized to glyceraldehyde 3-phosphate dehydrogenase (GAPDH), a glycolysis enzyme [52], using the ΔΔCT method [53].

### 2.9. Spectral Analysis

Crude mitochondria were isolated as described above after growth in the presence or absence of Cm. Mitochondrial pellets were suspended in 1 mL of SEMP. Spectral analysis was performed using a modified version of a previously described procedure [54]. To lyse the mitochondria an equal volume of 10 mM MOPS, pH 7.2 containing 5% sodium deoxycholate was added. The sample was gently mixed and clarified by centrifugation in an Eppendorf tabletop centrifuge at top speed (13,000 rpm) for 5 min. The supernatant was removed and two cuvettes were filled with equal amounts of the mitochondrial solution. To oxidize the reference sample, a few crystals of potassium ferricyanide were added to the cuvette. The sample in the other cuvette was reduced with a few crystals of sodium dithionite. The samples were placed in a Biochrom Ultrospec 3100 pro spectrophotometer (GE Healthcare, Little Chalfont, UK) and the absorbance was measured continuously from 650 to 500 nm to obtain the oxidized vs. reduced spectrum. Original graphs were traced with a pencil and scanned into Adobe Photoshop.

### 2.10. General Procedures and Other Techniques

Standard procedures were followed for DNA agarose gel electrophoresis, transformation of *Escherichia coli*, cloning, restriction digests of plasmid DNA, and site directed PCR mutagenesis of plasmid DNA [46]. Bacterial plasmid DNA was isolated using Qiagen mini-prep column spin kits (Qiagen Inc., Santa Clarita, CA, USA) following the supplier’s instructions. Sodium dodecyl sulphate polyacrylamide gel electrophoresis (SDS-PAGE) [55] and Western blotting [56] were performed as previously described. Samples for SDS-PAGE were prepared in cracking buffer (0.06 M Tris-HCl, pH 6.8; 2.5% SDS; 5% sucrose; 5% β-mercaptoethanol). Unless otherwise specified, all lanes on SDS-PAGE gels contained 30 μg protein. Western blot detection was performed using the LumiGLO chemiluminescent substrate system (Mandel Scientific Company Inc., Guelph, ON, Canada). Protein concentration was determined using the colourimetric Bio-Rad protein assay kit (Bio-Rad, Hercules, CA, USA). For automated sequencing, a BigDye Terminator cycle sequencing kit (V. 3.1) was used. The reactions were analyzed using a model 3730 DNA Analyzer (Applied Biosystems, Foster City, CA, USA) in the Molecular Biology Service Unit (MBSU) of the Department of Biological Sciences, University of Alberta.

### 2.11. Antibody to S. cerevisiae RPL3

Antibody to the *S. cerevisiae* ribosomal protein RPL3 was obtained from the Developmental Studies Hybridoma Bank (DSHB) and was originally deposited there by J.R. Warner (DSHB hybridoma product ScRPL3).

## 3. Results

### 3.1. Attempts to Rescue Single Deletion Mutant Strains by Transformation

As described in the introduction, we previously identified mutants in the *N. crassa* single gene deletion library [38] that were characterized as class 1 mutants because they were severely deficient in their ability to produce the AOD1 protein [29]. The deletion in eight of these mutants affected genes not previously known to be involved in production of AOD1. In the present study, we investigated these eight new mutants to determine more precisely their role in AOD1 production. The mutants were named according to the single gene deletion library plate number followed by the grid location on that plate [29,38]. The eight class I mutants that were under investigation in the current study included 23H2, 40E6, 41G7, 47H10, 52D8, 83H3, 88H8, and 100B5. We also included an additional mutant, 113G8, that was found to have the characteristics of a class I mutant after our initial analysis of the deletion library was published. Thus, a total of nine mutants were under investigation.

We began by attempting to rescue strain 100B5, which contains a deletion of the NCU08158 gene. Our eventual goal was to determine the intracellular location of the protein encoded by the deleted gene as a first step in attempting to relate its function to AOD1 production. Therefore, the rescue transformation was done using tagged versions of the gene. As described in the Materials and Methods, plasmids carrying resistance to basta and versions of the NCU08158 gene with a 3xHA tag at either the N- or C-terminus were constructed and transformed into strain 100B5. Selection and purification of transformants was done on medium containing basta as described in the Methods. Once purified strains for each transformation were obtained, they were tested for their ability to grow in the presence of AntA, which would indicate restoration of the ability to produce the AOD1 protein. Rescued transformants of strain 100B5 were obtained with both the N- and C-terminal 3xHA tagged versions of NCU08158. These rescued strains were examined for their ability to produce AOD1 protein following growth in the presence of Cm. One N-tagged and one C-tagged strain, which produced levels of AOD1 similar to controls, were chosen for further work.

We also attempted to rescue strains 23H2 and 88H8 using a similar approach with the appropriately tagged wild type genes. However, these attempts were unsuccessful. Since it was previously noted that strains in the single gene deletion library may contain secondary mutations in addition to the deletion of the target gene [57,58], we performed genetic analysis on strains 23H2 and 88H8, plus the other six class 1 strains for which rescue had not been attempted (40E6, 41G7, 47H10, 52D8, 83H3, and 113G8). The mutant strains were crossed with a strain of opposite mating type (either NCN251 *A* or 76-26 *a*) that was wild type for AOD1 production and therefore resistant to AntA growth inhibition. Given that the library deletions were generated by replacing each target gene with a Hyg resistance cassette, the Hyg resistance and AntA sensitivity of each deletion strain, should always segregate together. Similarly, the Hyg sensitivity and AntA resistance of the wild type parent should segregate together. Thus, no Hyg resistant AntA resistant, or Hyg sensitive AntA sensitive recombinant progeny should be obtained if the only gene affected in a given deletion strain was the original gene targeted for deletion. However, crosses of the eight strains showed that only strain 40E6 produced no recombinant progeny while the other seven strains did produce recombinants (Table S1). These results demonstrated that undefined mutations in other genes were responsible for the AntA sensitivity and the lack of AOD1 that was observed in these seven strains. The most obvious genes to consider for the location of these mutations were the well characterized *aod-1*, *aod-2*, and *aod-5* genes. We sequenced the *aod-1* gene from all seven of the strains, using PCR products produced from the genomic DNA from each strain. Five strains contained the wild type sequence. However, strains 23H2 and 88H8 carried a cytosine insertion in the coding region of the *aod-1* gene at an identical position. This insertion results in a frame shift at codon 237 of the 363 amino acid coding sequence and produces a stop codon 31 codons downstream (Figure S1). Since our AOD1 antibody is specific for the last 122 amino acid residues of the protein [19], any truncated protein that was produced would not have been detected when the strains were examined for AOD1 content in our initial screen [29].

### 3.2. The NCU08158 Gene Encodes a YVH1 Protein

As described above, we successfully rescued strain 100B5 with HA-tagged versions of the NCU08158 gene that was deleted in the strain, demonstrating that deletion of this gene resulted in the AOD1 deficiency. The NCU08158 gene is 1461 bp long, contains one intron, and encodes a 438 amino acid, 47.2 kDa protein (NCBI XP_959650.1). BLAST analysis revealed that the protein shares 32% identity (E value = 3 × 10^−47^, BLASTP) with a tyrosine protein phosphatase from *S. cerevisiae* called Yvh1 (yeast vaccinia virus VH1 homolog), which contains a dual specificity phosphatase (DUSP) domain (NCBI NP_012292.3). The *N. crassa* protein also has 29% sequence identity (E value = 8 × 10^−26^) with the DUSP12 of *Homo sapiens* (NCBI NP_009171.1). An alignment of the three proteins is shown in Figure S2A. All three proteins contain the DUSP domain with the active site HCX_5_R motif in their N-terminal regions. In addition, the proteins have a conserved C-terminal domain, containing one His and seven Cys residues, which has been shown to bind two zinc atoms [59,60,61]. The YVH1 protein with this bipartite structure has been well conserved through evolution. We will refer to the *N. crassa* protein as YVH1 and to the gene as *yvh1*. The general structures of the *N. crassa* gene and protein are shown in Figure S2B.

### 3.3. Temporal Expression of AOD1 in the Δyvh1 Mutant

**Figure 1:**
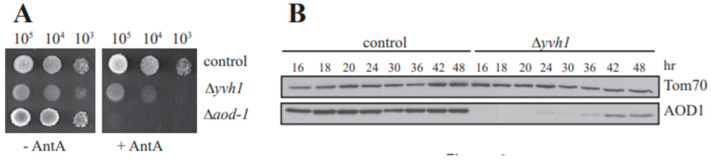
Growth of Δ*yvh1* cells and induction of AOD1. (**A**) Growth rate of Δ*yvh1*. Ten fold serial dilutions of conidia (numbers indicated at top of panel) from a wild type positive control strain (NCN251), a negative control strain (Δ*aod-1*) and Δ*yvh1* were spotted onto plates and incubated at 30 °C for 48 h on standard growth medium (−AntA) and for 68 h on medium containing AntA (+AntA). (**B**) Western blot analysis for the appearance of AOD1. Mitochondrial fractions were isolated from the control strain (NCN251) and Δ*yvh1* after growth in the presence of Cm for the specified times. Proteins were separated by SDS-PAGE, blotted to nitrocellulose membrane and probed with the antibodies indicated on the right. TOM70 [62], an outer mitochondrial membrane protein, was used as a loading control.

The Δ*yvh1* strain exhibits slightly reduced growth on medium without inhibitors of the electron transport chain, but a more obvious defect is observed when AntA is present in the medium (Figure 1A). The Δ*aod-1* strain, included as a negative control, is devoid of growth on AntA containing medium. However, the Δ*yvh1* mutant does exhibit some growth on AntA-containing plates with sorbose added (which forces colony formation). However, Δ*yvh1* did not grow at all on the non-sorbose, sucrose-containing plates (where linear mycelial growth occurs) with AntA that were used to quantify growth rates in our initial screen [29]. The reason for this difference in growth pattern is unknown. In our initial analysis we also found that the AOD1 protein was virtually undetectable after 24 h growth in liquid cultures of Δ*yvh1* containing the AOD1 inducing agent Cm, but was observed after 48 h of growth in the same medium [29]. Here, we further examined the production of AOD1 over time in the presence of Cm. The control strain contained AOD1 at all time points examined (Figure 1B). The Δ*yvh1* mutant contained little to no AOD1 for the first 20 h of growth. At 24 h a small amount of AOD1 was detectable in the mutant and at longer growth times a higher level of AOD1 was found. However, the amount present never reached the levels seen in the control over the 48 h growth period examined (Figure 1B).

### 3.4. Reduced Transcription of aod-1 in the Δyvh1 Mutant

**Figure 2:**
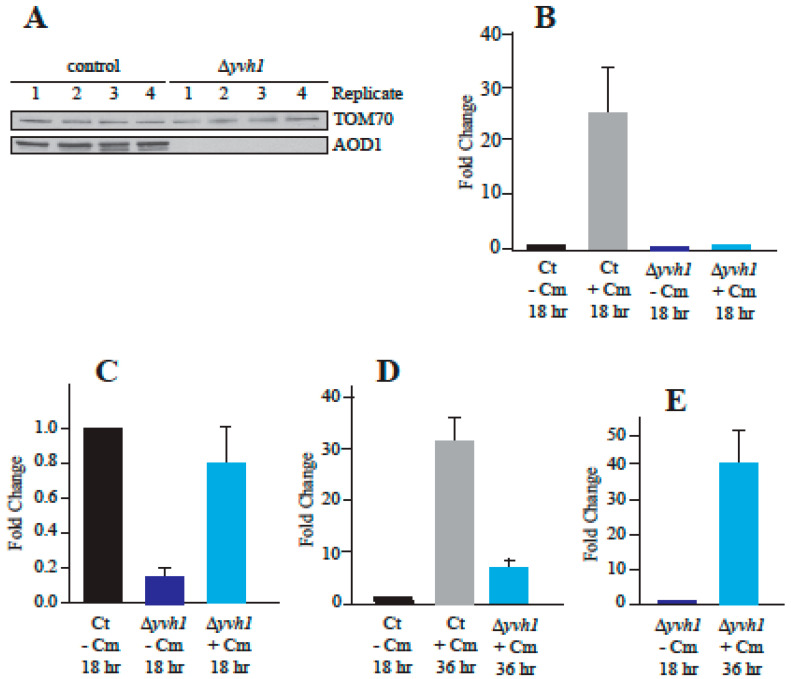
Levels of *aod-1* transcript in Δ*yvh1*. (**A**) Western blot analysis of biological replicates of the control strain (NCN251) and Δ*yvh1* grown in the presence of Cm for 18 hr. This was done as a control to insure that the 18 h mutant samples contained little to no detectable AOD1 protein prior to qPCR analysis. Mitochondria were isolated from the replicates and mitochondrial proteins were separated by SDS-PAGE, blotted to nitrocellulose membrane, and probed with the antibodies indicated on the right. RNA was also isolated from each replicate for use in qPCR analysis. (**B**–**E**) qPCR of *aod-1* transcript levels in control (Ct, NCN251) and Δ*yvh1* grown in the presence or absence of the inducer Cm (+Cm or −Cm) for 18 h or 36 h as indicated. In each panel the fold increase or decrease is shown relative to the first data set in that panel. Black bars indicate the control strain grown in the absence of Cm while grey bars indicate the control grown in the presence of Cm. Dark blue bars show the Δ*yvh1* mutant grown in the absence of Cm and light blue bars indicate the mutant grown in the presence of Cm. Error bars represent 95% confidence intervals. Fold change values were relative to the control (NCN251) grown in the absence of Cm for 18 h (**B**–**D**) or Δ*yvh1* grown in the absence of Cm for 18 h (**E**). Samples were normalized to GAPDH based on the ΔΔCT-method.

The production of the AOD1 protein in *N. crassa* is known to be controlled at the level of transcription of the *aod-1* gene [19,30,31,33,37] and at the level of translation of the transcript [19,37]. To distinguish between the two possible mechanisms preventing expression of the protein in Δ*yvh1* cells, we analyzed the levels of *aod-1* transcript in the deletion mutant. RNA was isolated from the deletion strain and a control, following 18 h growth in the presence or absence of Cm. qPCR was performed on the isolated mRNAs to determine the levels of the *aod-1* transcript. To confirm that no AOD1 protein was present in the mutant cultures grown in Cm, mitochondria were also isolated from the biological replicates of both the control and Δ*yvh1*. As predicted from the previous results (Figure 1B), after 18 h of growth in the presence of Cm, mitochondria from the Δ*yvh1* strain contained no detectable AOD1 protein while the control strain did (Figure 2A). The qPCR analysis showed that when the control strain was grown for 18 h in inducing conditions (+Cm), the *aod-1* transcript levels increased about 25 fold compared to the control without Cm (Figure 2B). When the Δ*yvh1* strain was grown for 18 h with or without Cm, transcript levels of *aod-1* were severely reduced, relative to the corresponding control, in both conditions (Figure 2B). To better illustrate the difference between the control and Δ*yvh1*, the data were replotted relative to the uninduced control using a smaller y-axis (Figure 2C). This plot shows that *aod-1* mRNA levels in uninduced mutant cells are about 10 fold lower in the mutant than in the uninduced control. The induced mutant cultures contained about seven fold more *aod-1* mRNA than the uninduced mutant. Thus, there was an increase in *aod-1* transcript levels in the Δ*yvh1* cells when grown in Cm (Figure 2C). However, the level in the induced mutant cultures is still slightly below that of the uninduced control and severely below the level of the induced control (Figure 2B,C). Taken together, these data show that reduced production of *aod-1* transcript accounts for the deficiency of AOD1 in the mutant strain. The data also show that in the 18 h Δ*yvh1* culture grown without Cm, the basal level of *aod-1* transcription is reduced. In the Δ*yvh1* cells grown in the presence of Cm, some induction occurs, but this falls well below the level of that observed in control cells grown under similar conditions. Thus, both the basal level of *aod-1* transcription and the ability to respond to the inducing signal are deficient in Δ*yvh1* cells.

To determine if the level of *aod-1* transcripts increased in Δ*yvh1* in older cultures, when AOD1 protein levels begin to rise, control and mutant cells were grown for 36 h in the presence of Cm. In the control strain, the level of expression after 36 h is about 30 fold higher than in the 18 h culture lacking Cm (Figure 2D), which is similar to the level in the 18 h control culture with Cm (Figure 2B). For Δ*yvh1*, the level of *aod-1* transcript in the 36 h plus Cm culture increased roughly six fold over the level in the 18 h minus Cm control culture (Figure 2D). The level of change between 18 h Δ*yvh1* minus Cm and 36 h Δ*yvh1* plus Cm was about 40 fold (Figure 2E). This increase probably accounts for the occurrence of detectable AOD1 protein in older Δ*yvh1* cultures grown in the presence of Cm (Figure 1B), even though it is still at reduced levels relative to the induced control after either 18 or 36 h.

### 3.5. Analysis of Cytochrome Spectra for Efficiency of Cm Inhibition

**Figure 3:**
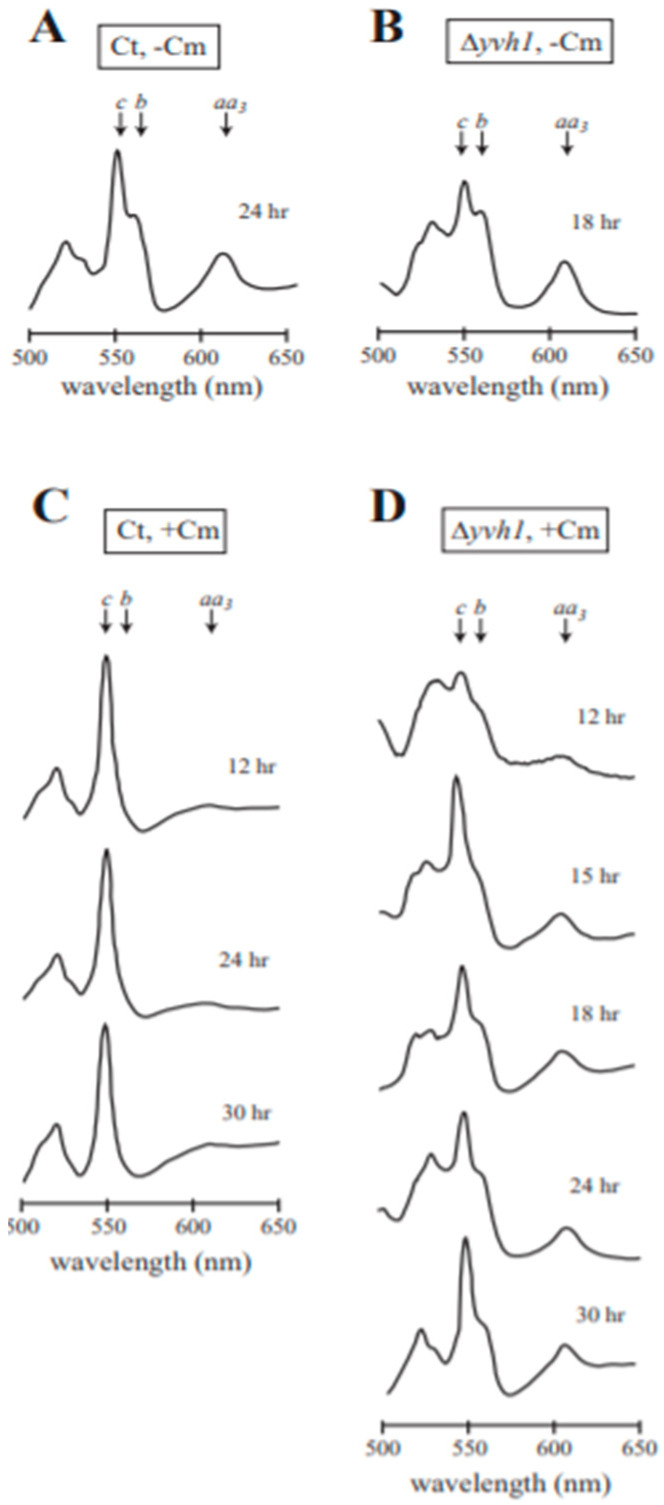
Cytochrome spectra. Qualitative cytochrome spectra of the NCN251 control (Ct) strain and the Δ*yvh1* strain, each grown in the absence (**A**,**B**) and presence (**C**,**D**) of Cm. The cytochrome content of mitochondria isolated from each culture was determined by scanning a reduced sample against an oxidized reference from 650 nm to 500 nm. The peaks indicated on the spectrum correspond to the absorbance maxima of cytochromes *aa*_3_ (605 nm), *b* (560 nm) and *c* (550 nm). The time of growth is indicated for each spectrum.

It was conceivable that the reduced production of AOD1 in Δ*yvh1* was due to inefficient inhibition of mitochondrial protein synthesis by Cm in the mutant strain, relative to the efficient inhibition by Cm in wild type cells. This would allow the sETC to function to a greater extent than in wild type cultures grown in the presence of Cm. The possibility was investigated by performing a qualitative examination of mitochondria for the spectrally detectable components of the electron transport chain, cytochromes *aa*_3_, *b*, and *c*. Mitochondrial translation products include cytochrome *b* of complex III and three proteins of cytochrome *aa*_3_, the spectral component of complex IV of the sETC [63]. Cytochrome *c* consists of a single polypeptide that is encoded in the nucleus, translated on cytosolic ribosomes, and imported into the mitochondria [64,65,66,67], and is therefore unaffected by the action of Cm, which inhibits mitochondrial protein synthesis. When grown under non-inducing conditions, mitochondria from wild type cells contained distinguishable peaks for cytochrome *c*, cytochrome *b*, and cytochrome *aa*_3_ as shown for the control strain NCN251 (Figure 3A). A similar pattern of these cytochromes was observed in mitochondria of the Δ*yvh1* strain grown in the absence of Cm (Figure 3B). As observed previously [23,28], when the wild type control was grown in the presence of Cm, the peaks for cytochrome *b* and cytochrome *aa*_3_ were severely reduced due to the decreased formation of mitochondrial translation products, while the nuclear encoded, cytosolically translated cytochrome c, was still present (Figure 3C). When the Δ*yvh1* strain was grown in the presence of Cm, the peaks for cytochrome *aa*_3_ and cytochrome *b* were somewhat reduced relative to the amount of cytochrome *c* (Figure 3D), but not to the extent observed in the control cultures grown in the presence of Cm (compare Figure 3C,D). These observations suggest that Cm may be affecting Δ*yvh1* mitochondrial translation to a lesser extent than in the control. This could help explain the slow accumulation of the AOD1 protein in the Δ*yvh1* mutant (Figure 2B) since more time might be required to accumulate a sufficient concentration of an unknown inducing signal for *aod-1* expression that accumulates when sETC function is decreased.

### 3.6. Characterization of Δyvh1 Transformants

**Figure 4:**
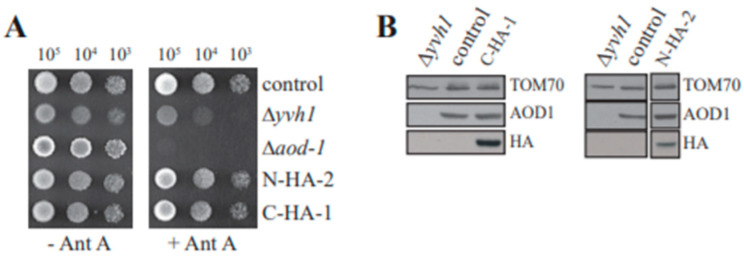
Transformants of Δ*yvh-1* expressing the 3xHA-tagged versions of YVH1 have growth rates and AOD1 expression similar to wild type cells. (**A**) Growth of strains N-HA2 and C-HA-1. Serial dilutions of conidia (indicated at top of panel) from the indicated strains were spotted on plates and incubated at 30 °C for 48 h on standard medium (−AntA) or 68 h on medium containing AntA (+AntA). The control strain is NCN251. (**B**) Western blot analysis of 3xHA-tagged strains. Cultures were grown in the presence of Cm for 20 hr. Crude mitochondrial and cytosolic fractions were prepared from the indicated strains. Proteins were separated by SDS-PAGE, and blotted to nitrocellulose membranes. The crude cytosolic fractions were probed with HA antibody. The mitochondrial fractions were probed with antibodies to AOD1 and Tom70 (loading control). Irrelevant lanes from the right portion of the panel were removed electronically.

As described above, we obtained AntA resistant colonies following transformation of strain 100B5 using plasmids carrying either the N- or C-terminal 3X HA-tagged versions of the NCU08158 gene, which encodes the YVH1 protein. Growth and localization studies were performed on both an N-terminal tagged transformant (N-HA-2) and C-terminal tagged transformant (C-HA-1). Analysis of both strains was done to minimize the possibility of artifactual observations arising from the location of the tag on the protein. The growth rate of both transformants was restored to control levels in both the absence and presence of AntA (Figure 4A). Examination of whole cell extracts from cultures of each strain grown in the presence of Cm for 20 h showed that the HA tag was detectable in both strains at a position corresponding to the predicted molecular size for *N. crassa* YVH1 including the 3xHA tag (50.4 kDa). Examination of mitochondria demonstrated that AOD1 levels were restored to wild type levels in cultures grown in the presence of Cm for 20 h (Figure 4B).

**Figure 5:**
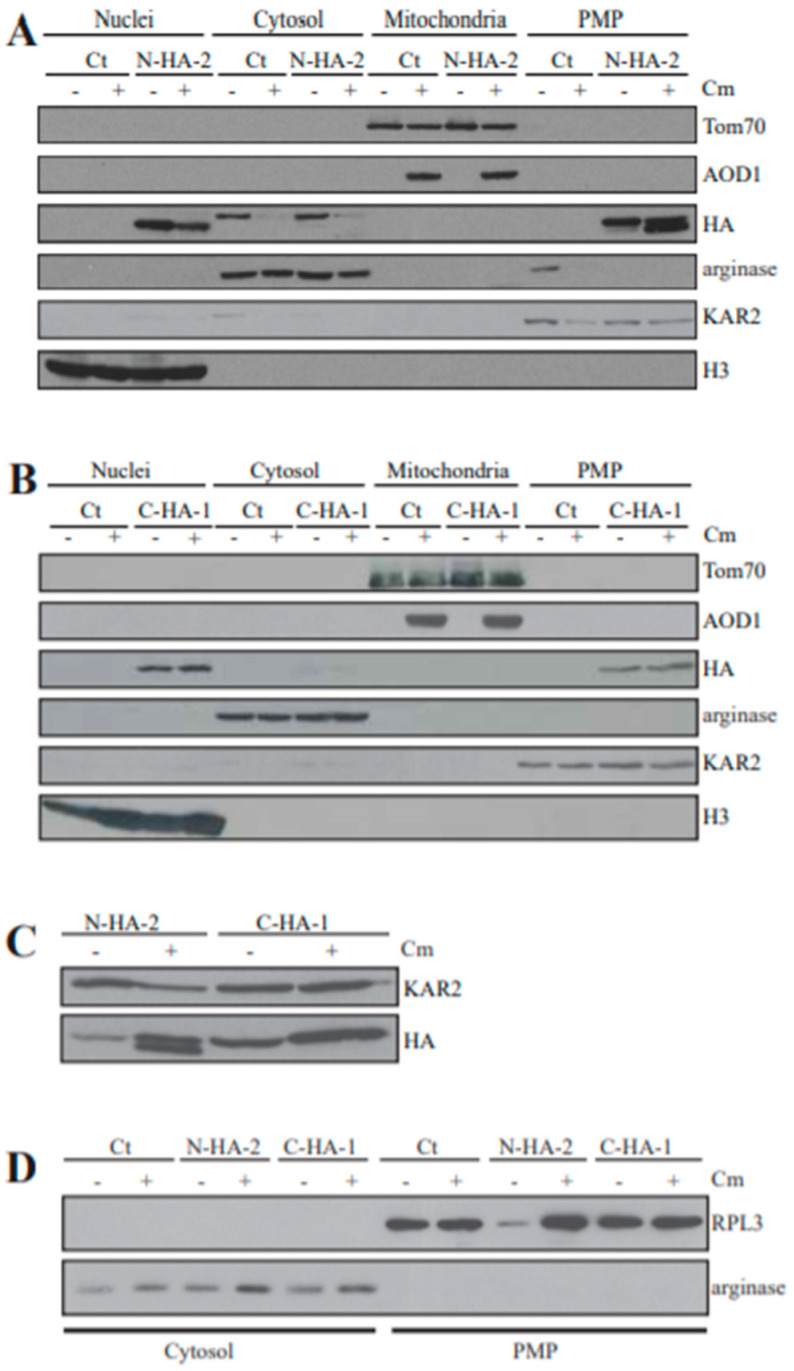
Subcellular localization of the YVH1 protein. (**A**,**B**) Western blot analysis of subcellular fractionation experiments from N- and C- terminal 3xHA tagged YVH1 strains (N-HA-2 and C-HA-1, respectively) and the control (ct) strain NCN251. Nuclei, cytosol, mitochondria, and Post-Mitochondrial Pellet (PMP) fractions were isolated from the N-HA-2 (**A**) and C-HA-1 (**B**) tagged YVH1 transformants as well as the control strain (NCN251) as described in the Material and Methods. Cultures were grown in the absence (−) or presence (+) of Cm for 20 h. Proteins were separated by SDS-PAGE, blotted to nitrocellulose membrane and probed with the antibodies indicated on the right. Antibodies that served as indicators to identify the different fractions were Tom70, as a mitochondrial marker [62]; arginase as a cytosolic marker [68]; the ER protein KAR2 as a microsomal marker in the PMP [69]; and Histone 3 (H3) as a nuclear marker. Antibody against the HA tag detects the YVH1 protein. (**C**) Comparative Western blot analysis of the HA tagged bands in the PMP fraction of the N-HA-2 and C-HA-1 transformants. PMP fractions were isolated from N-HA-2 and C-HA-1 grown in the absence (−) or presences (+) of Cm. Proteins were separated by SDS-PAGE, blotted to a nitrocellulose membrane, and probed with the antibodies indicated on the right. (**D**) Ribosomal proteins are found in the PMP fraction. Western blot analysis of cytosol and PMP fractions from the fractionations shown in panels A and B. Proteins were separated by SDS-PAGE, blotted to nitrocellulose membrane and probed with the antibodies indicated on the right. An *S. cerevisiae* RPL3 antibody was used as a ribosomal protein marker. Ct, control strain NCN251.

We then isolated subcellular fractions from N-HA-2 and C-HA-1 following growth in the presence and absence of Cm for 20 h. Fractions were examined for the presence of the HA tag as well as various marker proteins. Both the N-terminal and C-terminal tagged versions of YVH1 were present in both the nuclear and PMP fractions (Figure 5A,B). The PMP would be expected to contain fragmented ER and other organelles. The HA-tagged protein levels in strains N-HA-2 and C-HA-1 in both the PMP and nuclear fractions appeared to be roughly equal under both inducing and non-inducing conditions (Figure 5A,B). A band detected by the HA antibody was also occasionally seen in the cytosol of the strains grown in the absence of Cm (Figure 5A). We consider this to be a non-specific band since it also appeared in the non-HA tagged control strain and is slightly larger in molecular size than the HA specific band seen in other fractions.

The N-terminal tagged version of YVH1 that was present in the PMP fraction appeared as two bands of slightly different molecular size following growth in Cm (Figure 5A). Direct comparison of the N- and C-tagged proteins of the PMP fraction on the same gel confirmed that the larger molecular size protein detected in the N-terminal tagged strain is the same size as the protein from the C-terminal tagged strain (Figure 5C). This suggests that the protein is susceptible to a limited proteolytic cleavage at its C-terminus. It is unknown if the C-terminal tag prevents this proteolysis or if the shortened protein is simply undetectable once the HA tag is removed from the C-terminal tagged version of the protein.

It has been shown in both yeast and human culture cells that the YVH1 protein associates with ribosomal particles and plays a role in ribosome assembly (See Discussion). These observations, and the finding that a large amount of the *N. crassa* YVH1 was present in the PMP (Figure 5A,B) led us to ask if our PMP isolation protocol, using centrifugation, led to the pelleting of ribosomal particles. An antibody to *S. cerevisiae* RPL3, which has 75% identity with its *N. crassa* ortholog, was obtained from the Developmental Studies Hybridization Bank. Our PMP and cytosol (i.e., the post PMP supernatant) fractions were examined with this antibody as well as a marker for the cytosol. As shown in Figure 5D, it appears that ribosomal subunits were present in the PMP but not the cytosol, as was the tagged YVH1 (Figure 5A,B). Thus the presence of YVH1 in our PMP is likely due to association with ribosomal particles. This is in agreement with previous findings in *S. cerevisiae* [40].

### 3.7. AOX Expression Requires a Functional Zinc Binding Domain but Not the Phosphatase Domain of YVH1

**Figure 6:**
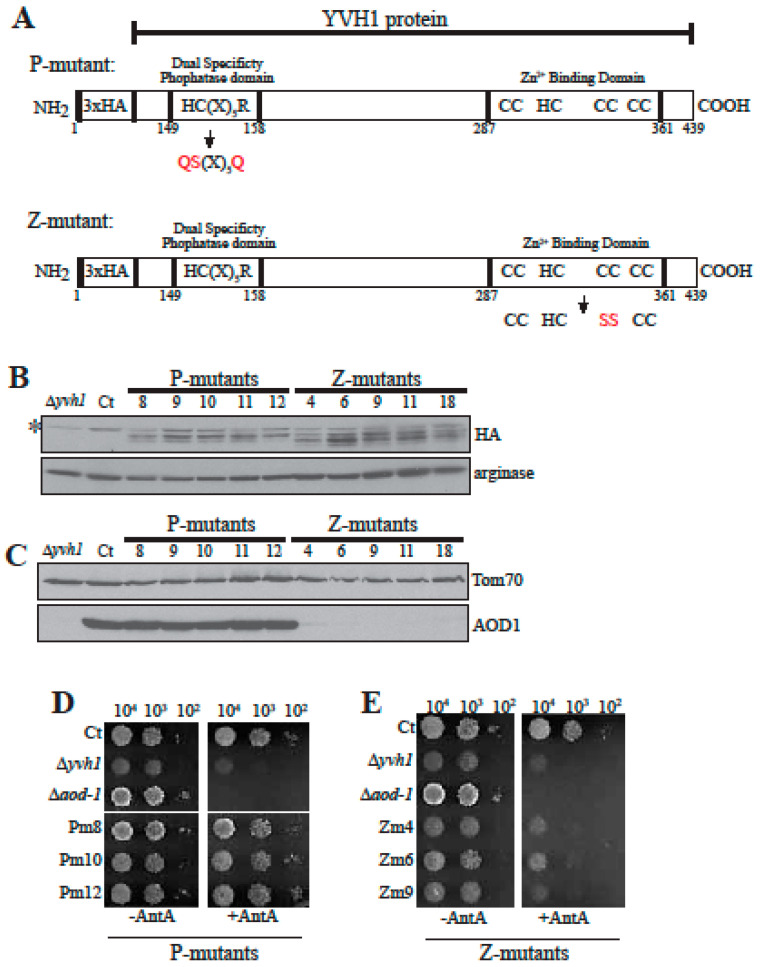
Analysis of phosphatase domain (P-mutants, Pm) and zinc binding domain (Z-mutants, Zm) mutants. (**A**) Representation of the *N. crassa* N-terminally 3xHA tagged YVH1 protein showing the position of amino acid changes introduced to create the P- and Z-mutants. The amino acid changes in the P-mutant are H149Q, C150S, and R156Q. The amino acid changes in the Z-mutants are C340S and C345S. The HA tag was not considered in the numbering of the amino acids. Changed amino acids are shown in red. (**B**) The 3xHA tagged mutant versions of YVH1 are expressed in isolated transformants of the Δ*yvh1* mutant. Whole cell extracts were prepared from the Δ*yvh1* strain, the NCN251 control (Ct), five isolates of P-mutant transformants, and five isolates of Z-mutant transformants. Cells were grown for 20 h in the presence of Cm. Proteins present in the cell extracts were separated by SDS-PAGE and blotted to nitrocellulose membranes. The blots were examined for HA and the loading control arginase. The * symbol indicates non-specific binding to an unknown protein by the HA antibody in the whole cell extract fractions. (**C**) Mitochondrial fractions were isolated from the same transformant strains expressing the Pm and Zm mutant versions of YVH1 that are shown in panel B. Strains were grown for 20 h in the presence of Cm. Proteins of the isolated mitochondria were separated by SDS-PAGE, blotted to nitrocellulose and probed with antibodies to AOD1, and Tom70 as the loading control. (**D**,**E**) Growth rate of selected N-terminal 3xHA tagged YVH1 P-mutants (Pm, **D**) and Z-mutants (Zm, **E**). Serial dilutions of conidia (indicated at the top of the panels) from the indicated strains were spotted on plates containing standard medium with (+AntA) and without AntA (−AntA). Plates without AntA were incubated at 30 °C for 48 h. Plates containing AntA were incubated at 30 °C for 68 h. Irrelevant rows were removed electronically. Ct, control strain NCN251. The Δ*yvh1* and Δ*aod-1* strains were also used as controls for growth rate.

YVH1 contains a DUSP domain that has been shown to have activity in vitro and is required for interaction with Hsp70 in human cells. The domain is also essential for protection from cell death induced by certain stress factors in human cells [60,70]. However, all examined negative phenotypes seen in *S. cerevisiae* mutants lacking *yvh1* can be restored to wild type by rescue with a gene encoding a protein containing the functional zinc-binding domain but lacking a functional phosphatase domain [39,40,41,60,61]. Since AOX is not produced in either *S. cerevisiae* or mammals, we wished to determine if either or both of the known domains of YVH1 were required for AOD1 expression in *N. crassa*. Mutants of each domain in the N-terminal 3xHA tagged version of YVH1 were created and transformed into the Δ*yvh1* strain. The phosphatase domain mutants (P-mutants or Pm) have the HC(X)_5_R dual specificity phosphatase domain changed to QS(X)_5_Q (Figure 6A, top). The zinc binding domain mutants (Z-mutants or Zm) have two of the Cys residues involved in zinc binding changed to Ser residues (C340S, C345S) (Figure 6A, bottom). The constructs containing these changes also contained a basta resistance gene and working isolates were selected for growth on medium containing basta. Colonies were picked, allowed to conidiate and streaked for single colony isolates on basta containing medium. Several isolates of each transformant were then examined for the presence of the HA-tagged protein and the ability to produce AOD1 when grown in the presence of Cm. All transformants examined were found to express the HA-tagged YVH1 protein (Figure 6B). The P-mutant transformants had normal levels of AOD1 after 20 h of growth in the presence of Cm, while the Z-mutants did not (Figure 6C). When three transformants of each type were tested for growth, the phosphatase domain mutants grew at the same rate as the wild type control on both standard medium and medium containing AntA (Figure 6D). However, the zinc domain mutants were not rescued and grew at the Δ*yvh1* rate on both types of medium (Figure 6E). These data demonstrated that the zinc-binding domain is required for proper growth and AOD1 expression, while the phosphatase domain is not.

### 3.8. Localization of Mutant YVH1 Proteins

**Figure 7:**
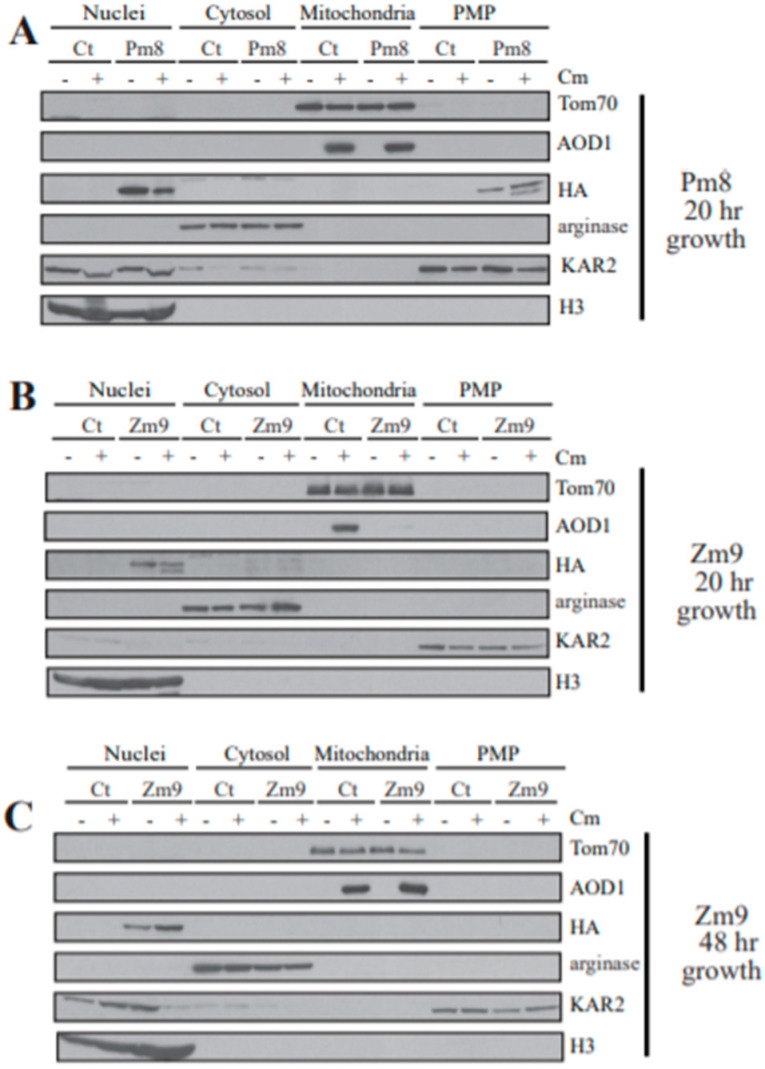
Subcellular fractionation of 3xHA N-terminal tagged P- and Z-mutant YVH1 strains. Nuclei, mitochondria, cytosol, and PMP fractions were isolated as described in the Materials and Methods from phosphatase domain mutant Pm8 (**A**) and from the zinc-binding domain mutant Zm9 (**B**,**C**) following growth for 20 h (**A**,**B**) or 48 h (**C**). The control (ct) strain was NCN251. Cultures were grown in the absence (−) or presence (+) of Cm. Proteins in each fraction were separated by SDS-PAGE, blotted to nitrocellulose membrane and probed with the antibodies indicated on the right. Markers for each fraction were as in the legend to Figure 5.

The P-mutant strain 8 (Pm8) and the Z-mutant strain 9 (Zm9) were then used in subcellular fractionation experiments to determine the localization of the altered protein. The mutant YVH1 protein in Pm8 was found in the nuclear and PMP fractions (Figure 7A), as were the N- and C-terminal HA tagged wild type versions of YVH1 (Figure 5A,B). AOD1 levels in the mitochondrial fraction were similar to the control when the cells were grown in the presence of Cm. These data reinforce the idea that YVH1 does not require a functional phosphatase domain to allow expression of AOD1 to control levels when cells are grown in the presence of Cm.

Subcellular fractionation of the zinc binding domain mutant Zm9, revealed that the protein only appears in the nuclear fraction following either 20 or 48 h growth in the presence or absence of Cm (Figure 7B,C). This suggests a possible role of the domain in shuttling the protein out of the nucleus. Also, after 20 h of growth in the presence of Cm, AOD1 was not detectable (Figure 7B), as is the case in Δ*yvh1*. After 48 h of growth, levels of AOX appeared to be at the control levels in the Zm9 mutant (Figure 7C). This was unexpected given our previous observation that AOD1 is detectable in the deletion mutant after 48 h of growth but does not reach the levels seen in controls (Figure 1B). This result suggested the possibility that in the absence of a functional Z-domain, the phosphatase domain might play a role in allowing full restoration of AOD1 levels at longer growth time in Cm. Alternatively, it is conceivable that the gradient purified mitochondria examined in Figure 7C have a different AOD1 concentration than the crude mitochondria analyzed in Figure 1B.

## 4. Discussion

This study was centered on uncharacterized mutants previously identified in the *N. crassa* single gene deletion library [38] that were severely deficient in production of AOD1 when grown under inducing conditions [29]. This included eight mutants from the original screen (23H2, 40E6, 41G7, 47H10, 52D8, 83H3, 88H8, and 100B5), plus an additional mutant (113G8,) identified following the publication of the initial screen. A combination of gene rescue experiments and genetic analysis revealed that the inability to produce AOD1 was due to the targeted gene deletion in only mutants 40E6 (NCU05600) and 100B5 (NCU08158). The other seven mutants contained secondary mutations that led to the AOD1 deficiency. The presence of secondary mutations in the *N. crassa* single gene deletion library has previously been reported during studies of mutants affecting hyphal cell fusion [57] and female development [58].

The location of the secondary mutations in the remaining seven AOD1 deficient strains seemed likely to be in one of the five genes now verified to affect AOD1 production. These include the *aod-1* structural gene, the transcription factors *aod-2* and *aod-5*, and the newly identified genes in strains 40E6 (NCU05600) and 100B5 (NCU08158 or *yvh1*). Sequencing of the *aod-1* gene in the seven strains carrying secondary mutations strains revealed an identical one nucleotide insertion in the *aod-1* gene of strains 23H2 and 88H8. This frame shift mutation would lead to production of a truncated AOD1 protein as the result of a premature stop codon (Figure S1). We do not know if the truncated protein is present in the mutant cells following growth in Cm, but it is not likely to be functional even if it is. Many of the C-terminal residues lost as a result of the truncation are known to play roles in important AOX functions including dimer formation, membrane binding, and coordinating the diiron center [6]. It is not clear how two of the strains in the deletion library, would have the same secondary mutation at the same position in the *aod-1* gene. Conceivably, a small proportion of the conidia used for transformations during creation of the deletion mutant library carried the *aod-1* mutation and those conidia were transformed in the generation of the deletions in 23H2 and 88H8.

For the remaining five secondary mutation strains, it is of note that in mutants 41G7 and 52D8 the AOD1 protein is reduced at 24 h and absent after 48 h growth in the presence of Cm. This is similar to the pattern observed in the verified 40E6 deletion mutant [29]. Thus, it is conceivable that 41G7 and 52D8 carry random mutations in the NCU05600 gene. Strains 47H10, 83H3 and 113G8 completely lack AOD1 following growth for either 24 or 48 h of growth in the presence of Cm. The *aod-2* and *aod-5* genes in these strains are candidates for the site of secondary mutations. Taken together, the results of these studies show that of the nine new class I mutants affecting AOD1 expression examined, only two (40E6 and 100B5) are actually due to the deletion of genes previously unknown to be involved with AOD1 production. We have not investigated the class 2 and 3 mutants found in our previous study. However, given that the AOD1 deficiency identified in 7 of 12 of the class 1 mutants was not due to deletion of the target gene, we would predict a similar ratio for the other mutant classes. Thus, it is highly recommended that genetic cross analysis be completed on any strains for which further work is considered.

Strain 100B5, which carries a deletion of the *yvh1* gene (NCU08158), was investigated further in the present study. In the Δ*yvh1* mutant, the AOD1 protein begins to appear in small amounts after about 24 h of growth in the presence of Cm and does not reach a level equal to the control even after 48 h of growth in Cm. This is a considerable delay compared to wild type *N. crassa* where the AOD1 protein has been observed after only 2.5 h of growth in the presence of AntA or Cm [19], and respiration via the AOD1 pathway has been observed after 30 min of exposure to Cm [71]. The low level of the AOD1 protein observed in the Δ*yvh1* strain following growth in Cm appears to be due to a low level of *aod-1* transcript. A slight increase in *aod-1* transcript level over time correlated to the appearance of the AOD1 protein. Previously we reported that Δ*yvh1* showed no growth after 48 h in the presence of AntA [29]. In the present study, the strain was allowed to grow for 63 h in the presence of AntA and a small amount of growth was observed. The delay in growth on AntA probably correlates to the delay in the appearance of the AOD1 protein as we observed following growth in the presence of Cm. Here, we also noted that the Δ*yvh1* strain has a slow growth phenotype on standard medium lacking inhibitors.

Rescued transformants of *N. crassa* Δ*yvh1* were obtained by introduction of *yvh1^+^* genes encoding proteins with 3xHA tags at their N- or C-terminus. Transformant strains were found to grow at control rates on medium with or without AntA, and to produce AOD1 protein at control levels following growth in the presence of Cm for 20 h. The tagged versions of the *N. crassa* YVH1 protein localized to the nuclear fraction and the PMP fraction in both inducing and non-inducing conditions. This pattern is similar to that found in *S. cerevisiae* and human cells where the protein has been shown to localize to the nucleus and the cytoplasm [39,40,41,60,70]. Furthermore, the majority of YVH1 in *S. cerevisiae* was found to be associated with ribosomal particles [40], which are present in our PMP fraction. It was also shown that YVH1 shuttled between the nucleus and cytoplasm in the malaria parasite, *P. falciparum* [72].

The *S. cerevisiae* Yvh1 protein was first identified as a homologue of the vaccinia virus VH1 protein. Both proteins carry a conserved phosphatase domain. The viral protein has the capacity to remove phosphate groups from both phosphoserine- and phosphotyrosine-containing proteins. The yeast protein was shown to act on a phosphotyrosine substrate in vitro but was not active on phosphoserine containing substrates [59]. Mutations in the phosphatase domain of the yeast protein have been shown to inactivate the in vitro activity [61]. The YVH1 protein also contains a zinc binding domain consisting of seven Cys residues and one His residue. The domain binds two mols of zinc per mol of protein [60]. Various phenotypes including reduced growth rate, sporulation defects, defects in glycogen accumulation, and problems with ribosome assembly have all been observed in *S. cerevisiae* mutants lacking the protein [39,40,41,42,59,61,73,74,75]. All these phenotypes can be rescued with mutant versions of the gene that encode Yvh1 proteins lacking the phosphatase activity, but containing the zinc binding domain. Rescue did not occur using genes in which the zinc binding domain was inactivated [39,40,41,60,61]. Thus, the role of the phosphatase domain in the *S. cerevisiae* protein remains unclear and its in vivo target is not known. Similar findings for the importance of the Zn binding domain in the rescue of multiple phenotypes in *yvh1* mutants of *Magnoporthe oryzae* have been reported [76].

The function of the human version of YVH1 (also known as DUSP12), has also been investigated. It was identified in a two-hybrid screen as a binding partner of glucokinase and has been shown to dephosphorylate this enzyme in vitro [77]. It has also been implicated in various cancers [78,79,80,81,82,83], and it plays a role in regulation of cell cycle progression [84]. Furthermore, an interaction with Hsp70 and a role for human YVH1 in protecting cells from external stresses has also been demonstrated [70]. For example, cell death resulting from either heat shock, exposure to hydrogen peroxide, or activation of the fas receptor to induce apoptosis, was reduced by overexpression of YVH1. The protective effect required both the phosphatase activity and the ability to interact with Hsp70, which required the zinc binding domain. The Cys residues in the zinc binding domain of human YVH1 have also been shown to play a role in providing a reversible redox sink that protects the Cys residue located in the phosphatase active site in times of oxidative stress [85].

To determine the role of the two domains on AOD1 expression in *N. crassa*, the phosphatase and zinc binding domains were mutated separately. When genes encoding YVH1 proteins with changes in the phosphatase domain were transformed into Δ*yvh1* cells, localization of the protein, growth rate of cells, and expression of the AOD1 protein all occurred as in wild type cells. On the other hand, when Δ*yvh1* cells were transformed with a gene encoding a YVH1 protein in which two Cys residues in the zinc-binding domain were changed to Ser residues, the transformants grew at the rate of Δ*yvh1* cells and did not produce AOD1 after 20 h growth in the presence of Cm. Therefore, *N. crassa* requires a YVH1 protein with a functional zinc-domain for the wild type pattern of AOD1 expression, but the phosphatase activity is not required.

Although there is a broad range of phenotypes reported for cells lacking the YVH1 protein in various organisms, the primary function of YVH1 appears to be in ribosome assembly. In *S. cerevisiae*, Yvh1 has been shown to associate with ribosomal subunits and to act as a factor required for the maturation of pre-60S ribosomal subunits [39,40,41,42]. Pre-60S subunits contain the protein Mrt4, which is homologous to the mature 60S subunit protein, P0. Yvh1 displaces Mrt4 from the maturing subunit and is subsequently released itself as P0 is assembled. In cells lacking Yvh1, Mrt4 is not released. This results in a delay in processing of pre-ribosomal RNA, a defect in exporting the pre-60S subunits from the nucleus, and inefficient assembly of the ribosome stalk [40,41,42]. Mrt4 suppressors have been found that bypass the need for YVH1. Interestingly, these Mrt4 suppressors also rescue the other phenotypes of *yvh1* mutants [40,41,42]. Thus, it seems likely that the other phenotypes are secondary manifestations of the ribosome assembly defect. Interactions of Yvh1 with other proteins required for 60S ribosome maturation have also been detected [86,87,88]. Recently, details of the role, structure, and point of action of Yvh1 in the process of ribosome assembly have been defined more precisely [89,90,91].

YVH1 appears to play a similar role in ribosomal subunit assembly in HeLa cells [40]. Furthermore, human YVH1 can rescue the slow growth phenotype of the *S. cerevisiae* deletion strain [60] and appears to fulfill the role of releasing Mrt4 from the yeast pre-60S subunit [40]. Thus, the ribosome assembly function of Yvh1 has been conserved through evolution. The human YVH1 has been shown to associate with ribosomal subunits and other ribonucleoprotein (RNP) particles, including stress granules [43]. We have not investigated ribosome assembly in the *N. crassa* mutant, but since this function of the protein has been conserved from yeast to humans, it seems likely that this is also the function of the protein in *N. crassa*.

Given that the primary function of YVH1 is in ribosome assembly, it is probable that the effects on AOD1 production are secondary. However, it is unclear exactly how and why this occurs. The effects appear to be at the level of transcription, or possibly mRNA stability since the level of transcripts is reduced in both induced and uninduced cultures of the Δ*yvh1* mutant. Conceivably the transcription factors for *aod-1* expression are reduced in amount or are less active in the strain. Effects on both expression of specific genes and on mRNA stability have been observed in *S. cerevisiae yvh1* mutants [42,73]

The expression of *aod-1* in *N. crassa* Δ*yvh1* cells may also be affected by the lack of a full inducing signal. The cytochrome spectral analyses revealed that inhibition of mitochondrial translation by Cm is not as effective in Δ*yvh1* as in the control. It is possible that the inducing signal resulting from Cm inhibition may not be present at sufficient levels to trigger expression of *aod-1*. However, if the signal accumulates over time, a partial induction of *aod-1* transcription may occur, followed by appearance of low levels of AOD1 protein. One possibility for a decreased effect of Cm might be that the drug does not accumulate in mitochondria of the mutant to the same extent as in controls. This might be due to overexpression of an efflux pump as another secondary effect of the inefficient ribosome assembly. It is also conceivable that unknown changes in the cell, resulting from the deletion of *yvh1*, lead to the strain becoming resistant to the effects of Cm. In conclusion, our investigation has shown that the production of the AOD1 protein in *N. crassa* is severely affected by lack of the YVH1 protein. Furthermore, the zinc binding domain of YVH1 is required for efficient AOD1 production, while the phosphatase domain is not. Work in other systems has shown that the role of YVH1 is in ribosomal assembly and it seems likely that this is the role of the protein in *N. crassa* as well. Since we have shown that the decreased production of AOD1 is due to a deficiency of *aod-1* transcripts, the actual mechanism by which this ribosomal assembly factor YVH1 specifically affects the level of the *aod-1* transcript remains unclear. As discussed above, determining the function and level of the *aod-1* transcription factors and the effectiveness of Cm in Δ*yvh1* cells offer possible routes of future investigation into this question.

## Figures and Tables

**Table 1:** Strains used in this study.

Strain	Comments	Origin
NCN251	Mating type *A*. Also called 74-OR23-1VA.	FGSC #2489
76-26	Mating type *a*. *his-3*, *mtr.*	R.L. Metzenberg
Δ*aod-1* (also known as 96H9)	Mating type *A. aod-1* gene replaced with a hygromycin resistance cassette.	*N. crassa* single gene deletion library. FGSC #18947
Δ*yvh1* (also known as 100B5)	Mating type *A*. *yvh1* gene replaced with a hygromycin resistance cassette.	*N. crassa* single gene deletion library. FGSC #19644
N-HA-2	Strain Δ*yvh1* with an ectopic copy of the genomic *yvh1* gene that also encodes an N-terminal 3xHA tag. Basta and hygromycin resistant.	This study
C-HA-1	Strain Δ*yvh1* with an ectopic copy of the genomic *yvh1* gene that also encodes a C-terminal 3xHA tag. Basta and hygromycin resistant.	This study
P-mutants 8, 9, 10, 11, and 12.	YVH1 phosphatase domain mutants. Obtained via transformation of Δ*yvh1* with an N-terminal 3xHA tagged version of *yvh1* that contained codons for amino acids H149, C150, and R156 in the HC(X)_5_R dual specificity phosphatase domain mutated to QS(X)_5_Q. Created by transformation of Δ*yvh1* with plasmid that also carried resistance to basta.	This study
Z mutants 4, 6, 9, 11, and 18.	YVH1 zinc binding domain mutants. Obtained via transformation of Δ*yvh1* with an N-terminal 3xHA tagged version of *yvh1* that contained codons for amino acids cysteine 340 and 345 of the zinc binding domain mutated to serine. Created by transformation of Δ*yvh1* with plasmid that also carried resistance to basta.	This study
23H2	NCU08887 replaced by Hyg^R^ cassette, mating type *A.*	*N. crassa* single gene deletion library. FGSC# 15957
40 E6	NCU05600 replaced by Hyg^R^ cassette, mating type *a.*	*N. crassa* single gene deletion library. FGSC# 13805
41G7	NCU03589 replaced by Hyg^R^ cassette, mating type *A.*	*N. crassa* single gene deletion library. FGSC# 13924
47H10	NCU00778 replaced by Hyg^R^ cassette, mating type *a.*	*N. crassa* single gene deletion library. FGSC# 16938
52D8	NCU07281 replaced by Hyg^R^ cassette, mating type *A.*	*N. crassa* single gene deletion library. FGSC# 14469
83H3	NCU08365 replaced by Hyg^R^ cassette, mating type *a.*	*N. crassa* single gene deletion library. FGSC# 18277
88H8	NCU01542 replaced by Hyg^R^ cassette, mating type *a.*	*N. crassa* single gene deletion library. FGSC# 19221
113G8	NCU03071 replaced by Hyg^R^ cassette, mating type *a.*	*N. crassa* single gene deletion library. FGSC# 18202

## Supplementary Materials

The following are available online at https://www.mdpi.com/2076-2607/8/8/1186/s1, Figure S1: A mutation exists in the aod-1 gene of deletion library strains 23H2 and 88H8, Figure S2: The N. crassa YVH1 gene and protein, Table S1: Analysis of crosses between wild type strains and class 1 AOD1 deficient strains.

## References

[1] Liu Z., Butow R.A. (2006). Mitochondrial retrograde regulation. Annu. Rev. Genet..

[2] Jazwinski S.M. (2013). The retrograde response: When mitochondrial quality control is not enough. Biochim. Biophys. Acta.

[3] da Cunha F.M., Torelli N.Q., Kowaltowski A.J. (2015). Mitochondrial retrograde signaling: Triggers, pathways, and outcomes. Oxid. Med. Cell. Longev..

[4] Cardamone M.D., Tanasa B., Cederquist C.T., Huang J., Mahdaviani K., Li W., Rosenfeld M.G., Liesa M., Perissi V. (2018). Mitochondrial retrograde signaling in mammals is mediated by the transcriptional cofactor GPS2 via direct mitochondria-to-nucleus translocation. Mol. Cell.

[5] Isaac R.S., McShane E., Churchman L.S. (2018). The multiple levels of mitonuclear coregulation. Annu. Rev. Genet..

[6] Moore A.L., Shiba T., Young L., Harada S., Kita K., Ito K. (2013). Unraveling the heater: New insights into the structure of the alternative oxidase. Annu. Rev. Plant. Biol..

[7] Shiba T., Kido Y., Sakamoto K., Inaoka D.K., Tsuge C., Tatsumi R., Takahashi G., Balogun E.O., Nara T., Aoki T. (2013). Structure of the trypanosome cyanide-insensitive alternative oxidase. Proc. Natl. Acad. Sci. USA.

[8] May B., Young L., Moore A.L. (2017). Structural insights into the alternative oxidases: Are all oxidases made equal?. Biochem. Soc. Trans..

[9] McDonald A.E., Vanlerberghe G.C. (2006). Origins, evolutionary history, and taxonomic distribution of alternative oxidase and plastoquinol terminal oxidase. Comp. Biochem. Physiol. Part D.

[10] McDonald A.E. (2008). Alternative oxidase: An inter-kingdom perspective on the function and regulation of this broadly distributed “cyanide-resistant” terminal oxidase. Funct. Plant. Biol..

[11] McDonald A.E. (2009). Alternative oxidase: What information can protein sequence comparisons give us?. Physiol. Plant..

[12] Neimanis K., Staples J.F., Huner N.P., McDonald A.E. (2013). Identification, expression, and taxonomic distribution of alternative oxidases in non-angiosperm plants. Gene.

[13] Joseph-Horne T., Holloman D.W., Wood P.M. (2001). Fungal respiration: A fusion of standard and alternative components. Biochim. Biophys. Acta.

[14] Vanlerberghe G.C., McIntosh L. (1997). Alternative oxidase: From gene to function. Annu. Rev. Plant. Physiol. Plant. Mol. Biol..

[15] Nargang F.E., Kennell J.C., Borkovich K.A., Ebbole D.J. (2010). Mitochondria and respiration. Cellular and Molecular Biology of Filamentous Fungi.

[16] Vanlerberghe G.C. (2013). Alternative oxidase: A mitochondrial respiratory pathway to maintain metabolic and signaling homeostasis during abiotic and biotic stress in plants. Int. J. Mol. Sci..

[17] Vanlerberghe G.C., Dahal K., Alber N.A., Chadee A. (2020). Photosynthesis, respiration and growth: A carbon and energy balancing act for alternative oxidase. Mitochondrion.

[18] Li Q., Ritzel R.G., McLean L.T.T., McIntosh L., Ko T., Bertrand H., Nargang F.E. (1996). Cloning and analysis of the alternative oxidase of *Neurospora crassa*. Genetics.

[19] Tanton L.L., Nargang C.E., Kessler K.E., Li Q., Nargang F.E. (2003). Alternative oxidase expression in *Neurospora crassa*. Fungal Genet. Biol..

[20] Lambowitz A.M., Slayman C.W. (1971). Cyanide-resistant respiration in *Neurospora crassa*. J. Bacteriol..

[21] Henry M.F., Nyns E.J. (1975). Cyanide-insensitive respiration: An alternative mitochondrial pathway. Subcell. Biochem..

[22] Lambowitz A.M., Sabourin J.R., Bertand H., Nickels R., McIntosh L. (1989). Immunological identification of the alternative oxidase of *Neurospora crassa* mitochondria. Mol. Cell. Biol..

[23] Bertrand H., Argan A., Szakacs N.A., Schweyen R.J., Wolf K., Kaudewitz F. (1983). Genetic control of the biogenesis of cyanide insensitive respiration in *Neurospora crassa*. Mitochondria 1983.

[24] Tissieres A., Mitchell H.K., Haskins F.A. (1953). Studies on the respiratory system of the *poky* strain of *Neurospora*. J. Biol. Chem..

[25] Lambowitz A.M., Smith E.W., Slayman C.W. (1972). Electron transport in *Neurospora* mitochondria: Studies on wild type and *poky*. J. Biol. Chem..

[26] Kuntzel H. (1969). Proteins of mitochondrial and cytoplasmic ribosomes from *Neurospora crassa*. Nature.

[27] McKee E.E., Ferguson M., Bentley A.T., Marks T.A. (2006). Inhibition of mammalian mitochondrial protein synthesis by oxazolidinones. Antimicrob Agents Chemother.

[28] Descheneau A.T., Cleary I.A., Nargang F.E. (2005). Genetic evidence for a regulatory pathway controlling alternative oxidase production in *Neurospora crassa*. Genetics.

[29] Nargang F.E., Adames K., Rub C., Cheung S., Easton N., Nargang C.E., Chae M.S. (2012). Identification of genes required for alternative oxidase production in the *Neurospora crassa* gene knockout library. G3.

[30] Chae M.S., Nargang C.E., Cleary I.A., Lin C.C., Todd A.T., Nargang F.E. (2007). Two zinc cluster transcription factors control induction of alternative oxidase in *Neurospora crassa*. Genetics.

[31] Chae M.S., Lin C.C., Kessler K.E., Nargang C.E., Tanton L.L., Hahn L.B., Nargang F.E. (2007). Identification of an alternative oxidase induction motif in the promoter region of the *aod-1* gene in *Neurospora crassa*. Genetics.

[32] Chae M.S., Nargang F.E. (2009). Investigation of regulatory factors required for alternative oxidase production in *Neurospora crassa*. Physiol. Plant..

[33] Qi Z., Smith K.M., Bredeweg E.L., Bosnjak N., Freitag M., Nargang F.E. (2017). Alternative oxidase transcription factors AOD2 and AOD5 of *Neurospora crassa* control the expression of genes involved in energy production and metabolism. G3.

[34] Sellem C.H., Bovier E., Lorin S., Sainsard-Chanet A. (2009). Mutations in two zinc cluster proteins activate alternative respiratory and gluconeogenic pathways and restore senescence in long-lived respiratory mutants of *Podospora Anserina*. Genetics.

[35] Suzuki Y., Murray S.L., Wong K.H., Davis M.A., Hynes M.J. (2012). Reprogramming of carbon metabolism by the transcriptional activators AcuK and AcuM in *Aspergillus nidulans*. Mol. Microbiol..

[36] Bovier E., Sellem C.H., Humbert A., Sainsard-Chanet A. (2014). Genetic and functional investigation of Zn2Cys6 transcription factors RSE2 and RSE3 in *Podospora anserina*. Eukaryot Cell.

[37] Bosnjak N., Smith K.M., Asaria I., Lahola-Chomiak A., Kishore N., Todd A.T., Freitag M., Nargang F.E. (2019). Involvement of a G protein regulatory circuit in alternative oxidase production in *Neurospora crassa*. G3.

[38] Colot H.V., Park G., Turner G.E., Ringelberg C., Crew C., Litvinkova L., Weiss R.L., Borkovich K.A., Dunlap J.C. (2006). A high-throughput gene knockout procedure for *Neurospora* reveals functions for multiple transcription factors. Proc. Natl. Acad. Sci. USA.

[39] Liu Y., Chang A. (2009). A mutant plasma membrane protein is stabilized upon loss of Yvh1, a novel ribosome assembly factor. Genetics.

[40] Lo K.Y., Li Z., Wang F., Marcotte E.M., Johnson A.W. (2009). Ribosome stalk assembly requires the dual-specificity phosphatase Yvh1 for the exchange of Mrt4 with P0. J. Cell Biol..

[41] Kemmler S., Occhipinti L., Veisu M., Panse V.G. (2009). Yvh1 is required for a late maturation step in the 60S biogenesis pathway. J. Cell Biol..

[42] Sugiyama M., Nugroho S., Iida N., Sakai T., Kaneko Y., Harashima S. (2011). Genetic interactions of ribosome maturation factors Yvh1 and Mrt4 influence mRNA decay, glycogen accumulation, and the expression of early meiotic genes in Saccharomyces cerevisiae. J. Biochem..

[43] Geng Q., Xhabija B., Knuckle C., Bonham C.A., Vacratsis P.O. (2017). The atypical dual specificity phosphatase hYVH1 associates with multiple ribonucleoprotein particles. J. Biol. Chem..

[44] Davis R.H., De Serres F.J. (1970). Genetic and microbiological research techniques for *Neurospora crassa*. Method. Enzym..

[45] Metzenberg R.L. (2003). Vogel’s medium N salts: Avoiding the need for ammonium nitrate. Fungal Genet. Newslett..

[46] Sambrook J., Russell D.W. (2001). Molecular Cloning: A Laboratory Manual.

[47] Pall M.L., Brunelli J.P. (1993). A series of six compact fungal transformation vectors containing polylinkers with multiple unique restriction sites. Fungal Genet. Newslett..

[48] Hoppins S.C., Go N.E., Klein A., Schmitt S., Neupert W., Rapaport D., Nargang F.E. (2007). Alternative splicing gives rise to different isoforms of the *Neurospora crassa* Tob55 protein that vary in their ability to insert β-barrel proteins into the outer mitochondrial membrane. Genetics.

[49] Nargang F.E., Rapaport D., Leister D.L., Herrmann J. (2007). Neurospora crassa as a model organism for mitochondrial biogenesis. Mitochondria. Practical Protocols.

[50] Lambowitz A.M. (1979). Preparation and analysis of mitochondrial ribosomes. Methods Enzymol..

[51] Talbot K.J., Russell P.J. (1982). Nuclear buoyant density determination and the purification and characterization of wild-type neurospora nuclei using percoll density gradients. Plant. Physiol..

[52] Nicholls C., Li H., Liu J.P. (2012). GAPDH: A common enzyme with uncommon functions. Clin. Exp. Pharm. Physiol..

[53] Livak K.J., Schmittgen T.D. (2001). Analysis of relative gene expression data using real-time quantitative PCR and the 2^−ΔΔCT^ method. Methods.

[54] Bertrand H., Pittenger T.H. (1969). Cytoplasmic mutants selected from continuously growing cultures of *Neurospora crassa*. Genetics.

[55] Laemmli U.K. (1970). Cleavage of structural proteins during the assembly of the head of bacteriophage T4. Nature.

[56] Towbin H., Staehelin T., Gordon J. (1979). Electrophoretic transfer of proteins from polyacrylamide gels to nitrocellulose sheets: Procedure and some applications. Proc. Natl. Acad. Sci. USA.

[57] Fu C., Iyer P., Herkal A., Abdullah J., Stout A., Free S.J. (2011). Identification and characterization of genes required for cell-to-cell fusion in *Neurospora crassa*. Eukaryot Cell.

[58] Chinnici J.L., Fu C., Caccamise L.M., Arnold J.W., Free S.J. (2014). *Neurospora crassa* female development requires the PACC and other signal transduction pathways, transcription factors, chromatin remodeling, cell-to-cell fusion, and autophagy. PLoS ONE.

[59] Guan K., Hakes D.J., Wang Y., Park H.D., Cooper T.G., Dixon J.E. (1992). A yeast protein phosphatase related to the vaccinia virus VH1 phosphatase is induced by nitrogen starvation. Proc. Natl. Acad. Sci. USA.

[60] Muda M., Manning E.R., Orth K., Dixon J.E. (1999). Identification of the human YVH1 protein-tyrosine phosphatase orthologue reveals a novel zinc binding domain essential for in vivo function. J. Biol. Chem..

[61] Beeser A.E., Cooper T.G. (2000). The dual-specificity protein phosphatase Yvh1p regulates sporulation, growth, and glycogen accumulation independently of catalytic activity in *Saccharomyces cerevisiae* via the cyclic AMP-dependent protein kinase cascade. J. Bacteriol..

[62] Grad L., Descheneau A., Neupert W., Lill R., Nargang F. (1999). Inactivation of the *Neurospora crassa* mitochondrial outer membrane protein TOM70 by repeat-induced point mutation (RIP) causes defects in mitochondrial protein import and morphology. Curr. Genet..

[63] Attardi G., Schatz G. (1988). The biogenesis of mitochondria. Annu. Rev. Cell Biol..

[64] Korb H., Neupert W. (1978). Biogenesis of cytochrome c in mitochondria: Synthesis of apocytochrome *c*, transfer to mitochondria and conversion to holocytochrome *c*. Eur. J. Biochem..

[65] Pfanner N., Neupert W. (1990). The mitochondrial protein import apparatus. Annu. Rev. Biochem..

[66] Bottorff D.A., Parmaksizoglu S., Lemire E.G., Coffin J.W., Bertrand H., Nargang F.E. (1994). Mutations in the structural gene for cytochrome c result in deficiency of both cytochromes *aa*_3_ and *c* in *Neurospora crassa*. Curr. Genet..

[67] Neupert W., Herrmann J. (2007). Translocation of proteins into mitochondria. Annu. Rev. Biochem..

[68] Borkovich K.A., Weiss R.L. (1987). Purification and characterization of arginase from *Neurospora crassa*. J. Biol. Chem..

[69] Addison R. (1998). A cell-free translation-translocation system reconstituted with subcellular fractions from the wall-less variant *fz; sg*; *ox-1V* of *Neurospora crassa*. Fungal Genet. Biol..

[70] Sharda P.R., Bonham C.A., Mucaki E.J., Butt Z., Vacratsis P.O. (2009). The dual-specificity phosphatase hYVH1 interacts with Hsp70 and prevents heat-shock-induced cell death. Biochem. J..

[71] Edwards D.L., Rosenberg E., Maroney P.A. (1974). Induction of cyanide-insensitive respiration in *Neurospora crassa*. J. Biol. Chem..

[72] Kumar R., Musiyenko A., Cioffi E., Oldenburg A., Adams B., Bitko V., Krishna S.S., Barik S. (2004). A zinc-binding dual-specificity YVH1 phosphatase in the malaria parasite, *Plasmodium falciparum*, and its interaction with the nuclear protein, pescadillo. Mol. Biochem. Parasitol..

[73] Park H.D., Beeser A.E., Clancy M.J., Cooper T.G. (1996). The *S. cerevisiae* nitrogen starvation-induced Yvh1p and Ptp2p phosphatases play a role in control of sporulation. Yeast.

[74] Beeser A.E., Cooper T.G. (1999). The dual-specificity protein phosphatase Yvh1p acts upstream of the protein kinase mck1p in promoting spore development in *Saccharomyces cerevisiae*. J. Bacteriol..

[75] Sakumoto N., Mukai Y., Uchida K., Kouchi T., Kuwajima J., Nakagawa Y., Sugioka S., Yamamoto E., Furuyama T., Mizubuchi H. (1999). A series of protein phosphatase gene disruptants in *Saccharomyces cerevisiae*. Yeast.

[76] Liu X., Qian B., Gao C., Huang S., Cai Y., Zhang H., Zheng X., Wang P., Zhang Z. (2016). The putative protein phosphatase MoYvh1 functions upstream of MoPdeH to regulate the development and pathogenicity in *Magnaporthe oryzae*. Mol. Plant Microbe Interact..

[77] Munoz-Alonso M.J., Guillemain G., Kassis N., Girard J., Burnol A.F., Leturque A. (2000). A novel cytosolic dual specificity phosphatase, interacting with glucokinase, increases glucose phosphorylation rate. J. Biol. Chem..

[78] Hirai M., Yoshida S., Kashiwagi H., Kawamura T., Ishikawa T., Kaneko M., Ohkawa H., Nakagawara A., Miwa M., Uchida K. (1999). 1q23 gain is associated with progressive neuroblastoma resistant to aggressive treatment. Genes Chromosomes Cancer.

[79] Gratias S., Schuler A., Hitpass L.K., Stephan H., Rieder H., Schneider S., Horsthemke B., Lohmann D.R. (2005). Genomic gains on chromosome 1q in retinoblastoma: Consequences on gene expression and association with clinical manifestation. Int. J. Cancer.

[80] Mendrzyk F., Korshunov A., Benner A., Toedt G., Pfister S., Radlwimmer B., Lichter P. (2006). Identification of gains on 1q and epidermal growth factor receptor overexpression as independent prognostic markers in intracranial ependymoma. Clin. Cancer Res..

[81] Biernacki M.A., Marina O., Zhang W., Liu F., Bruns I., Cai A., Neuberg D., Canning C.M., Alyea E.P., Soiffer R.J. (2010). Efficacious immune therapy in chronic myelogenous leukemia (CML) recognizes antigens that are expressed on CML progenitor cells. Cancer Res..

[82] Nguyen le B., Diskin S.J., Capasso M., Wang K., Diamond M.A., Glessner J., Kim C., Attiyeh E.F., Mosse Y.P., Cole K. (2011). Phenotype restricted genome-wide association study using a gene-centric approach identifies three low-risk neuroblastoma susceptibility loci. PLoS Genet..

[83] Cain E.L., Braun S.E., Beeser A. (2011). Characterization of a human cell line stably over-expressing the candidate oncogene, dual specificity phosphatase 12. PLoS ONE.

[84] Kozarova A., Hudson J.W., Vacratsis P.O. (2011). The dual-specificity phosphatase hYVH1 (DUSP12) is a novel modulator of cellular DNA content. Cell Cycle.

[85] Bonham C.A., Vacratsis P.O. (2009). Redox regulation of the human dual specificity phosphatase YVH1 through disulfide bond formation. J. Biol. Chem..

[86] Sakumoto N., Yamashita H., Mukai Y., Kaneko Y., Harashima S. (2001). Dual-specificity protein phosphatase Yvh1p, which is required for vegetative growth and sporulation, interacts with yeast pescadillo homolog in *Saccharomyces cerevisiae*. Biochem. Biophys. Res. Commun..

[87] Oeffinger M., Leung A., Lamond A., Tollervey D. (2002). Yeast pescadillo is required for multiple activities during 60S ribosomal subunit synthesis. RNA.

[88] Tarassov K., Messier V., Landry C.R., Radinovic S., Serna Molina M.M., Shames I., Malitskaya Y., Vogel J., Bussey H., Michnick S.W. (2008). An in vivo map of the yeast protein interactome. Science.

[89] Sarkar A., Pech M., Thoms M., Beckmann R., Hurt E. (2016). Ribosome-stalk biogenesis is coupled with recruitment of nuclear-export factor to the nascent 60S subunit. Nat. Struct. Mol. Biol..

[90] Zhou Y., Musalgaonkar S., Johnson A.W., Taylor D.W. (2019). Tightly-orchestrated rearrangements govern catalytic center assembly of the ribosome. Nat. Commun..

[91] Klingauf-Nerurkar P., Gillet L.C., Portugal-Calisto D., Oborska-Oplova M., Jager M., Schubert O.T., Pisano A., Pena C., Rao S., Altvater M. (2020). The GTPase Nog1 co-ordinates the assembly, maturation and quality control of distant ribosomal functional centers. Elife.

