# Supplementary material for: Characterization of Single Gene Deletion Mutants Affecting Alternative Oxidase Production in Neurospora crassa: Role of the yvh1 Gene
Source: Microorganisms. 2020 Aug 4;8(8):1186. doi: 10.3390/microorganisms8081186 (PMC7463738; doi:10.3390/microorganisms8081186)
Supplement: Supplementary file 1 [file microorganisms-08-01186-s001.pdf]

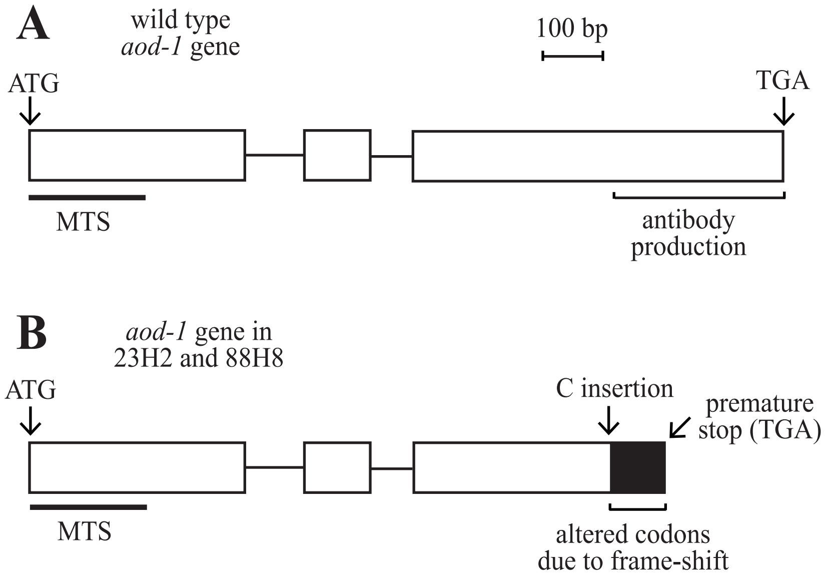

Supplementary Figure 1. A mutation exists in the *aod-1* gene of deletion library strains 23H2 and 88H8. A) The wild type version of the *aod-1* gene and B) the mutant version found in strains 23H2 and 88H8 are represented along with features of the gene and coding sequence. The cytosine (C) insertion in the *aod-1* gene of the mutants occurs 870 bp after the translation start site in the sequence including introns, or 708 bp from the translation start in the mature mRNA. The region of the protein used for antibody production is shown under the wild-type sequence. Open rectangular boxes, exons; thin black lines, introns; black box, frame-shift altered codons ending with a premature stop codon; thick black lines, MTS (mitochondrial targeting signal).

A

```
NcYVH1   --MALNRINGREDLFVGGIFG------VNRARLIEENKITHILSVINYTLPADPAFRNVQ 52
ScYVH1   MAGNANSVDEEVTRILGGIYLGGIRPIIDHRPLGAEFNITHILSVIKFQVIPEYLIRKGY 60
HsYVH1   MLEAPGPSDGCELSNPSASRVSCAGQMLEVQPGLYFGGAAAVAEPDHLREAGITAVLTVD 60
              .  :       ..         ::          : : .  :        . .

NcYVH1   HLS-IDIDDVEEADILVHFPKMVRFIERGLYGDEAASIAFGGDNAPVVVPTPASASAKIN 111
ScYVH1   TLKNIPIDDDDVTDVLQYFDETNRFIDQCLFPNEVEYSPR-------------------- 100
HsYVH1   SEEPSFKAGPGVEDLWRLFVPALDKPETDLLSHLDRCVAFIG------------------ 102
           .     .    *:   *       :  *  .     .

NcYVH1   GDDETAHDADAITAAPPITTTTITSQQQQTKPGAVLVHCAMGKSRSVSAIVAYLLWKHPH 171
ScYVH1   ----------------------LVDFKKKPQRGAVFAHCQAGLSRSVTFIVAYLMYRYG- 137
HsYVH1   --------------------------QARAEGRAVLVHCHAGVSRSVAIITAFLMKTDQ- 135
                                   : :.:  **:.**  * ****: *.*:*:

NcYVH1   RFGRSDPSTPARRAVTQAINWVRQTRPIAEPNDGFMEQLELWWTMGCPLESGDDAVENHP 231
ScYVH1   ------------LSLSMAMHAVKRKKPSVEPNENFMEQLHLFEKMG-----GDFVDFDNP 180
HsYVH1   ------------LPFEKAYEKLQILKPEAKMNEGFEWQLKLYQAMG------YEVDTSSA 177
                      ..  * . ::  :* .: *:.*  **.*:  **        .  . .

NcYVH1   AYQRWLYKREVEDATRIG-RVPDWIRFEDEEAAKLASENNNKEAEAGGGAASLSLRCKKC 290
ScYVH1   AYKQWKLKQSIKLDPSGSELVSNSGMFKDSESSQDLDKLTEAEKSK-----VTAVRCKKC 235
HsYVH1   IYKQYRLQKVTEKYPELQNLPQELFAVDPTTVSQGLKD-------------EVLYKCRKC 224
          *:::  ::  :  .       :   ..    ::  ..                 :*:**

NcYVH1   RRTLATKPFIVPHHQGK---------------------GNKERDCGHYFVEALSWMRPTL 329
ScYVH1   RTKLALSTSFIAHDPPSKESSEGHFIKRAANSHRIIDIQESQANCSHFFIEPLKWMQPEL 295
HsYVH1   RRSLFRSSSILDHREGSGPIAFAHKRMTPSS----MLTTGRQAQCTSYFIEPVQWMESAL 280
         * .*  .. :: *   .                        : :*  :*:*.:.**.. *

NcYVH1   E-QGELEGRLTCPNQKCLASVGRYTWQGFRCSCGDWIAPAFSLQKSKVDEATSAPHPGGA 388
ScYVH1   QGKQELEGKFSCPG--CSSKVGGYNWKGSRCSCGKWVIPAIHLQTSKVDQFPLQST---- 349
HsYVH1   L--GVMDGQLLCPK--CSAKLGSFNWYGEQCSCGRWITPAFQIHKNRVDEMKILPVLG-- 334
              ::*:: **   * :.:* :.* * :**** *: **: ::..:**:    .

NcYVH1   GRGPGGGGGGAAAVAARMAALGIRMPPGGLAGQRGAGEGAGAAGGPKENL 438
ScYVH1   ----------ALPNMVNFESEKVNR------------------------- 364
HsYVH1   -----------------SQTGKI--------------------------- 340
                            :  :
```

B

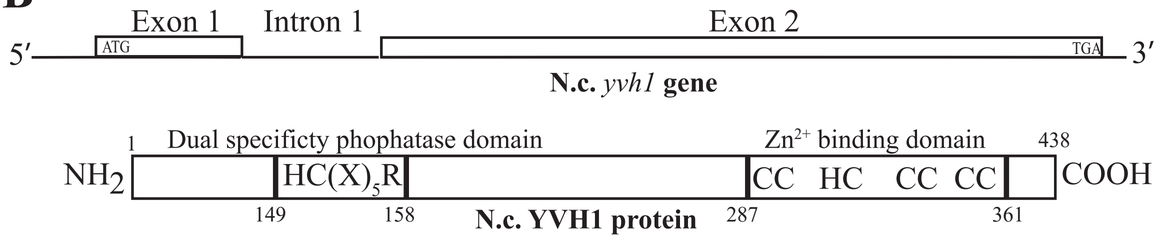

Supplementary Fig. 2. The *N. crassa* YVH1 gene and protein. A) The amino acid sequence of the YVH1 protein of *N. crassa* was aligned to the YVH1 proteins of *Saccharomyces cerevisiae* and *Homo sapiens* using the online ClustalW tool of the European Bioinformatics Institute. The amino acids that make up the dual specificity phosphatase domain in the N-terminal region are outlined in the black box. The seven Cys and one His that make up the zinc-binding domain in the C-terminal region are shaded in yellow. The "*" shows identical residues. The ":" shows conserved substitution of residues with the same physiochemical properties. "." shows semi-conserved substitution between residues in all three proteins. B) The *yvh1* gene of *N. crassa* contains 2 exons separated by 1 intron. The protein encoded is 438 amino acids long. The location of the HC(X)$_5$R dual specificity phosphatase domain and the zinc-binding domain containing seven Cys and one His residues are indicated.

Supplementary Table 1. Analysis of crosses between wild type strains and class 1 AOD1 deficient strains.

Phenotypes and numbers of ascospore progeny (% of those examined)					
	Parental phenotype		Recombinant phenotype		
Strain crossed	$Hyg^R AntA^S$	$Hyg^S AntA^R$	$Hyg^R AntA^R$	$Hyg^S AntA^S$	Number of progeny examined
23H2 *A*[1]	12 (19%)	15 (23%)	14 (22%)	23 (36%)	64
40E6 *a*	21 (36%)	38 (64%)	0 (0%)	0 (0%)	59
41G7 *A*	9 (9%)	44 (44%)	46 (45%)	2 (2%)	101
47H10 *a*	6 (9%)	52 (58%)	30 (33%)	0 (0%)	90
52D8 *A*	8 (12%)	53 (81%)	3 (5%)	1 (2%)	65
83H3 *a*	5 (6%)	55 (60%)	31 (34%)	0 (0%)	91
88H8 *a*	12 (24%)	12 (24%)	12 (24%)	14 (28%)	50
113G8 *a*	17 (21%)	54 (65%)	11 (13%)	1 (1%)	83

[1] Strains with mating type *A* were crossed with the 76-26 *a* strain. Strains with *a* mating type were crossed with NCN251 *A*. Both 76-26 and NCN251 are wild type with respect to AOD1 expression. In all crosses, the wild type strains were used as the female parent. The superscripts R and S indicate resistance or sensitivity, respectively, to the indicated drugs.
